# Trends and uncertainties in budburst projections of Norway spruce in Northern Europe

**DOI:** 10.1002/ece3.3476

**Published:** 2017-10-22

**Authors:** Cecilia Olsson, Stefan Olin, Johan Lindström, Anna Maria Jönsson

**Affiliations:** ^1^ Department of Physical Geography and Ecosystem Science Lund University Lund Sweden; ^2^ Centre for Mathematical Sciences Lund University Lund Sweden

**Keywords:** Bayesian inference, climate models, International Phenological Gardens, phenology models, *Picea abies*, provenance

## Abstract

Budburst is regulated by temperature conditions, and a warming climate is associated with earlier budburst. A range of phenology models has been developed to assess climate change effects, and they tend to produce different results. This is mainly caused by different model representations of tree physiology processes, selection of observational data for model parameterization, and selection of climate model data to generate future projections. In this study, we applied (i) Bayesian inference to estimate model parameter values to address uncertainties associated with selection of observational data, (ii) selection of climate model data representative of a larger dataset, and (iii) ensembles modeling over multiple initial conditions, model classes, model parameterizations, and boundary conditions to generate future projections and uncertainty estimates. The ensemble projection indicated that the budburst of Norway spruce in northern Europe will on average take place 10.2 ± 3.7 days earlier in 2051–2080 than in 1971–2000, given climate conditions corresponding to RCP 8.5. Three provenances were assessed separately (one early and two late), and the projections indicated that the relationship among provenance will remain also in a warmer climate. Structurally complex models were more likely to fail predicting budburst for some combinations of site and year than simple models. However, they contributed to the overall picture of current understanding of climate impacts on tree phenology by capturing additional aspects of temperature response, for example, chilling. Model parameterizations based on single sites were more likely to result in model failure than parameterizations based on multiple sites, highlighting that the model parameterization is sensitive to initial conditions and may not perform well under other climate conditions, whether the change is due to a shift in space or over time. By addressing a range of uncertainties, this study showed that ensemble modeling provides a more robust impact assessment than would a single phenology model run.

## 1 INTRODUCTION

Compared with the global average, climate warming is expected to be higher during winter months, and more pronounced further north and in mountainous regions, such as in the Alps (IPCC, 2013). Plant spring phenology is highly tuned to winter and spring temperatures and is thus a good indicator of climate change. Many plants have responded to the recent warming by becoming active earlier in the year, but the climate change response varies among species and locations and depends on the time period considered (Ahas, Aasa, Menzel, Vg, & Scheifinger, 2002; Menzel & Fabian, 1999; Menzel et al., 2008). Potential implications that may follow from phenological shifts in trees include longer growing seasons, which may increase forest productivity (Richardson et al., 2010). However, as frost hardiness in spring is negatively related to growth activity (Westin, Sundblad, Strand, & Hällgren, 2000), an earlier onset of the growing season may increase the risks and severity of frost damage during late spring cold spells (Jönsson & Bärring, 2011). For commercially important species like Norway spruce, for which large differences in phenology traits exist among provenances, comprehensive cultivation research is carried out to identify traits favorable in a warmer climate (e.g., Skrøppa & Steffenrem, 2016; Westin et al., 2000).

Reliable phenology models are needed to improve the simulations of terrestrial biosphere models, for more robust projections of climate change impacts on, for example, forest productivity and plant–atmosphere interactions (Jeong, Medvigy, Shevliakova, & Malyshev, 2012; Migliavacca et al., 2012; Richardson et al., 2012). When modeling climate change impacts on tree phenology, uncertainties propagate from initial conditions (i.e., the observed system state at the start of the simulation), model classes (i.e., process representations), model parameters (i.e., parameterization), and boundary conditions (i.e., assumption about model forcing data) (Araújo & New, 2007). Budburst models vary in their representation of tree physiology processes, for example, how various requirements are attained by interacting photoperiod, chilling, and forcing temperatures. Chilling requirements are particularly difficult to quantify, as dormancy release cannot be readily observed (Linkosalo, Häkkinen, & Hänninen, 2006), although it may be correlated with blocking of plasmodesmata by callose (Singh, Svystun, AlDahmash, Jönsson, & Bhalerao, 2017).

Provenances of Norway spruce, adapted to different environmental conditions, differ in the temperature sums required to trigger bud development, and tree breeders have for a long time been selecting trees with high growth capacity and low risk of frost damage (Hannerz, 1999). The lack of provenance‐specific requirements in phenology models may impose bias and uncertainty in simulations across geographical and climatic gradients (Chuine, Belmonte, & Mignot, 2000; Kramer et al., 2017; Olsson, Bolmgren, Lindström, & Jönsson, 2013; Olsson & Jönsson, 2015). Adding model parameters may however lead to increased uncertainty as more factors have to be taken into account (Beven, 2009). Using observations of known provenances for model parameterization, the uncertainty related to genetic differences is removed, and the spatial variation in phenology can be assumed to mainly represent the effect of local climate conditions (Chen, 2013). Statistical inference can be applied to account for parameter uncertainties (Beven, 2009), and by recognizing that all models have shortcomings but still provide useful information, ensemble simulations over multiple initial conditions, model classes, model parameterizations, and boundary conditions can be carried out to assess trends and uncertainties. That is, whereas model limitations are traditionally overcome by building better models, ensemble simulations provide an alternative way to generate robust projections (Araújo & New, 2007).

**Figure 1:**
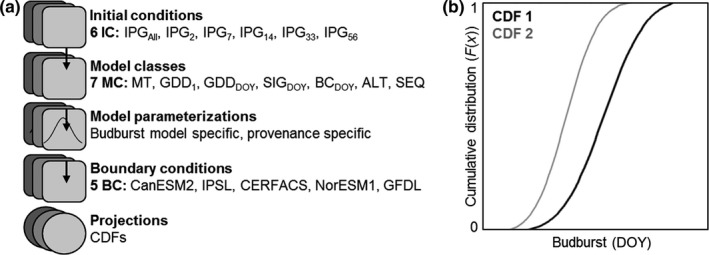
(a) The ensemble modeling scheme used in this study: Model simulations were produced for three Norway spruce provenance; one with an early timing of budburst originating from Germany (P121) and two with a late timing of budburst, originating from Germany (P122) and Norway (P123). Initial conditions (IC) for model parameterization came from observations at International Phenological Gardens (IPG); comparing the outcome of using data from all 23 IPGs with data from five single sites. Seven budburst models were successfully parameterized, representing a range of model classes (MC). Bayesian inference was applied for model parameterization. Model boundary conditions (BC) were provided by a subensemble of regional climate model data from SMHI‐RCA4, used in dynamical downscaling of five global climate models (CanESM2, IPSL CM5A MR, CERFACS CNRM CM5, NorESM1‐M, and GFDL‐GFDL ESM2M). The predictive distribution of each IC‐MC‐specific parameterization was sampled from the joint posterior distribution and forced with each BC, generating an ensemble consisting of 74,385 (P121), 71,705 (P122), and 76,235 (P123) model projections for each year and IPG. The simulations were aggregated into cumulative distribution functions (CDF), representing three time periods: 1971–2000 (TP1), 2011–2040 (TP2), and 2051–2080 (TP3). (b) Conceptual figure illustrating two CDFs with budburst projections, where CDF 1 represents past climate conditions (TP1) and CDF 2 future climate conditions (TP3) with earlier timing of budburst

The main objective of this study was to perform ensemble modeling of budburst in Norway spruce (*Picea abies*). The study region covered the main distribution area in Europe, north of the Alps, and the analysis included four categories of uncertainties, related to the following: (i) initial conditions (IC), (ii) model classes (MC), (iii) model parameterization, and (iv) boundary conditions (BC). The analysis of IC was carried out using phenology observations of three cloned provenances, grouped into six sets of observations used for model parametrization (Figure 1a). The analysis of MC included seven budburst models, varying in their representation of tree physiology processes, with each IC‐MC combination parameterized using Bayesian inference. The analysis of BC was carried out using a subensemble of five climate model datasets representing RCP8.5. Specifically, we assessed the following: (i) the provenance‐specific budburst projections for the study region, (ii) differences among budburst model projections, in relation to model representation of tree physiological processes, and (iii) the relative contribution of IC, MC, and BC to uncertainties in the model projections. Through this, we also assessed which budburst models were more challenging to parameterize in relation to observational constraints and initial inference values.

## 2 MATERIALS AND METHODS

### 2.1 Initial conditions

Observed timing of budburst was used for model parameterization and validation, representing the initial conditions in a climate change context. Data on budburst of Norway spruce (*Picea abies*) were provided by the network of International Phenological Gardens (IPG; Chmielewski, Heider, Moryson, & Bruns, 2013). Clones of known provenances have been planted in similar garden environments, mainly on plain meadows with sparse trees. Budburst is recorded by professional observers and has been defined as the first spring sprout when the buds open and the bud edges are visible with the needles not yet expanded (Chmielewski et al., 2013).

To correctly capture interannual variability and to separate differences in climatic response from local adaptation, it is preferable to use datasets with long time series. In this study, we therefore selected IPGs with more than 20 years of budburst records per Norway spruce provenance; a provenance from Germany with early timing of budburst (IPG plant no. 121, hereafter referred to as P121) and two provenances with late timing of budburst, one from Germany (P122) and one from northern Norway (P123). This generated a dataset of 23 IPGs and 1506 records from 1968 to 2013 (Table 1, Figure A1 in Appendix S1). Each time series was checked for outliers, using the 30‐day rule of Schaber and Badeck (2002). The five potential outliers identified were not removed from the analysis, considering the small deviations (between 30 and 36 days, Table 1) and the reliability of the records. The dataset was for each provenance grouped into six IC to constrain the model parameterization; parameterization across sites using observations from all IPGs (IPG_All_), and for comparison, site‐specific parameterization using five selected IPGs (IPG_2_, IPG_7_, IPG_14_, IPG_33,_ and IPG_56_). The selected IPG stations includes the station with the largest elevation difference between site and climate grid cell (IPG_2_), the most northern and eastern site (IPG_7_), most western site (IPG_14_), highest elevated site and with the latest budburst (IPG_33_), and the most southern and earliest budburst (IPG_56_).

**Table 1:** Summary of data on three Norway spruce provenances, one early (P121) and two late (P122, P123), at the International Phenological Gardens (IPG), with number of recorded budburst (*n*), average budburst (±*SD*), and location of the IPGs; latitude (Lat, °N), longitude (Lon, °E), altitude (Alt, m above sea level), the difference in altitude between the climate grid cell and the IPG site (Alt diff), and mean annual temperature (MAT) for the period with observations (1968–2013)

IPG ID, Name (Country code)		Lat	Lon	Alt	Alt dif	MAT (°C)	P121		P122		P123	
							*n*	Mean	*n*	Mean	*n*	Mean
1	Trondhjem‐Sjordal (NO)	63.3	10.53	70	109	4.9	31	143 ± 7	29	150 ± 5	35	150 ± 6
2	Bergen‐Fana (NO)	60.27	5.35	50	242	6.3	44	136 ± 10	44	142 ± 9	43	143 ± 11
7	Turku (FI)	64.52	26.45	115	19	2.0	47	147 ± 7	46	150 ± 6	46	150 ± 7
12	Aalborg (DK)	57.24	9.92	20	‐6	8.1	22	130 ± 5	22	141 ± 6	22	142 ± 7
14	Co‐Wexford (IE)	52.34	‐6.64	80	‐18	10.1	39	125 ± 9	41	127 ± 9	41^a^	125 ± 12
18	Gent‐Melle (IE)	50.98	3.80	15	16	10.1	26	118 ± 10	26^a^	121 ± 12	26	124 ± 11
23	Hannover‐Münden (D)	51.33	9.67	500	‐234	8.5	26	131 ± 9	25	140 ± 8	26	143 ± 10
24	Offenbach (D)	50.10	8.78	99	87	9.7	37	120 ± 8	27	123 ± 7	26	125 ± 10
26	Trier (D)	49.75	6.67	265	92	8.9	33	112 ± 8	33	125 ± 7	25	131 ± 7
27	Stuttgart‐Hohenheim (D)	48.72	9.22	380	‐26	9.5	27	114 ± 8	27	128 ± 7	46	127 ± 8
28	Stuttgart‐Weilimdorf (D)	48.82	9.12	330	24	9.5	31	125 ± 7	30	126 ± 6	29	124 ± 9
30	Kaiserstuhl‐Liliental (D)	48.07	7.68	265	131	9.1	40	118 ± 8	40	128 ± 8	41	126 ± 10
32	Freiburg‐Eschbach (D)	48.02	7.98	500	‐32	9.0	35	112 ± 11	35	126 ± 9	35	126 ± 8
33	Freiburg‐Schauinsland (D)	47.92	7.90	1210	‐443	7. 2	41	147 ± 11	41	153 ± 11	41	154 ± 11
36	München‐Grafrath (D)	48.18	11.17	540	56	8.3	44	133 ± 9	43	136 ± 10	31	137 ± 9
38	Freyung‐Schönbrunn (D)	48.85	13.52	737	3	6.4	33	133 ± 8	30	141 ± 9	27	140 ± 11
42	Tharandt‐Hartha (D)	50.98	13.53	360	73	7.2	46	128 ± 8	46	135 ± 8	46	137 ± 10
44	Mikolajki (PL)	53.78	21.58	127	17	7.1	38	131 ± 6	35	134 ± 7	32	135 ± 10
46	Zürich‐Birmensdorf (CH)	47.36	8.44	600	‐86	9.1	42^a^	120 ± 9	42^a^	129 ± 9	41^a^	128 ± 11
51	Slepcany‐Mlynany (SK)	48.33	18.37	180	47	9.8	40	120 ± 9	41	126 ± 7	41	129 ± 7
52	LVU‐Banska (SK)	48.45	18.93	540	92	7.1	39	125 ± 7	39	128 ± 8	38	133 ± 7
55	Ljubljana (SK)	46.07	14.50	310	136	9.5	46	112 ± 7	46	121 ± 7	45	120 ± 6
56	Zagreb (HR)	45.80	15.97	146	87	11.0	45	109 ± 9	45	116 ± 10	45	119 ± 10

a Outliers: P121; IPG_46_ (2012, 151), P122; IPG_18_ (1982, 88), IPG_46_ (2003, 94), P123; IPG_14_ (2006, 159), IPG_46_ (2003, 92).

### 2.2 Model classes

Budburst models range from being purely empirical to more process‐based in their representation of tree physiological processes. Previous studies indicate that models with few parameters in general have higher accuracy over larger regions than more complex models (Basler, 2016; Olsson & Jönsson, 2014) and that all models are less accurate when applied outside the range of conditions for which they are parameterized for (Olsson et al., 2013). To address uncertainties related to MC, we selected an ensemble of eight budburst models that have previously been applied across large regions; one empirical model and seven models based on temperature sums. The empirical model (MT), which is based on a linear regression between day of budburst and mean temperature in February to April (Table 2, Equation 1), was in a previous study among the better performing models for birch and Norway spruce in Europe (Olsson & Jönsson, 2014). The temperature sum models comprised of three varieties of a forcing model (Table 2, Equations 3–5), one forcing‐photoperiod model (Table 2, Equation 6), and three chilling–forcing models (Table 2, Equations 7–9). For all of these models, day of budburst was simulated to occur on the first day when the accumulated forcing exceeded the requirement (Table 2, Equation 2). Given that photoperiod can influence the timing of budburst, and thus protected from too early or too late budburst (Hänninen, 1990; Partanen, Koski, & Hänninen, 1998), we used August 1st as a cutoff threshold for model failure to simulating bud burst. During projection using climate model data, model failure was calculated as the percent of site and year combinations, for which a model was not able to predict budburst before August 1.

**Table 2:** Model equations

Equation	Model	Formula		
1	MT	$\text{obsDBB}=I+kT_{\mathrm{Feb}-\mathrm{Apr}}$		
2	All	$\text{simDBB}Sf_{t}\geq F_{\mathrm{crit}}$	Day of simulated budburst for all temperature sum models	
3	GDD_1_	$Sf_{t}=\sum_{t_{o}=\mathrm{jan}1}^{t}\left\{\begin{array}{cc} 0, & T_{t}<T_{b}\\ T_{t}-T_{b} & T_{t}\geq T_{b} \end{array}\right.$		
4	GDD_DOY_	$Sf_{t}=\sum_{t_{o}}^{t}\left\{\begin{array}{cc} 0, & T_{t}<T_{b}\\ T_{t}-T_{b} & T_{t}\geq T_{b} \end{array}\right.$		
5	SIG_DOY_	$Sf_{t}=\sum_{t_{0}}^{t}\frac{1}{1+e^{b(T_{c}+c)}}$		
6	BC_DOY_	$Sf_{t}=\sum_{t_{0}}^{t}\left\{\begin{array}{cc} 0, & T_{t}<T_{b}\\ \left(T_{t}-T_{b}\right)\times {\left(\frac{DL_{t}}{10}\right)}^{\mathrm{EXPO}}, & T_{t}\geq T_{b} \end{array}\right.$		
7	ALT	$Sc_{t}=\sum_{t_{2}}^{t}\left\{\begin{array}{cc} 0, & T_{t}\geq T_{b}\\ 1, & T_{t}<T_{b} \end{array}\right.$	$Sf_{t}=\sum_{t_{0}=\text{jan1}}^{t}\left\{\begin{array}{cc} 0, & T_{t}<T_{b}\\ T_{t}-T_{b} & T_{t}\geq T_{b} \end{array}\right.$	$F_{\mathrm{crit}}=\beta e^{-\gamma SC_{t}}$
8	SEQ	$Sc_{t}=\sum_{t_{2}}^{t}\left\{\begin{array}{cc} 0, & T_{t}\leq -3.4\;\text{or}\;T_{t}>10.4\\ \frac{T_{t}-\left(-3.4\right)}{T_{\mathrm{opt}}-\left(-3.4\right)}, & -3.4<T_{t}\leq T_{\mathrm{opt}}\\ \frac{T_{t}-10.4}{T_{\mathrm{opt}}-10.4}, & T_{\mathrm{opt}}<T_{t}\leq 10.4 \end{array}\right\}$	$Sf_{t}=\sum_{t_{0}=\text{jan1}}^{t}\left\{\begin{array}{cc} 0, & T_{t}<T_{b}\;\mathrm{and}\;Sc_{t}<C_{\mathrm{crit}}\\ \frac{1}{1+e^{b(Tc+c)}}, & T_{t}\geq T_{b}\;\mathrm{and}\;Sc_{t}\geq C_{\mathrm{crit}} \end{array}\right.$	
9	UNI	$Sc_{t}=\sum_{t_{2}}^{t}\frac{1}{1+e^{ac{\left(T_{c}-oC\right)}^{2}+bc\left(T_{t}-oC\right)}}$	$Sf_{t}=\sum_{t_{0}=\text{jan1}}^{t}\frac{1}{1+e^{dF(T_{t}-f_{F})}},Sc_{t}\geq C_{\mathrm{crit}}$	$F_{\mathrm{crit}}=\beta e^{-\gamma SC_{t}}$

Two of the forcing models include a linear response to temperatures above a base temperature (*T*_*b*_) (Landsberg, 1974); GDD_1_ from January 1 and GDD_DOY_ from a parameterized starting day. The other forcing model, SIG_DOY_, includes a sigmoidal temperature response for all temperatures from a parameterized starting day (Migliavacca et al., 2012). The forcing‐photoperiod model (BC_DOY_, Blümel & Chmielewski, 2012) is an extension of GDD_DOY,_ in which, longer days are associated with an enhanced temperature response, as defined by an exponential constant. The Alternating model (ALT, Cannell & Smith, 1983) is an extension of GDD_1_, with the forcing requirement exponentially reduced for each additional chilling day. The Sequential model (SEQ, Sarvas 1972 in Hänninen, 1990) is an extension of SIG, with the forcing temperature response conditioned on the break of winter rest, estimated to occur when a chilling requirement is reached. The most complex model applied in this study, the Unified model (UNI, Chuine, 2000), is an extension of SIG_1_, and share features with both SEQ (chilling requirement) and ALT (forcing requirement conditioned on the number of chilling days).

### 2.3 Model parameterization using Bayesian inference

The budburst models were parameterized using Bayesian inference for each provenance and initial condition separately. By propagating the parameter uncertainties into future projections, we account for the uncertainty in the estimated parameters, that is, different parameter values that yield similar predictions under current conditions may not do so under future conditions. To obtain the parameter values most likely given our observations (the posterior probability distribution, *p*(θ|IC)), Bayesian inference combines our prior beliefs regarding possible parameter values (the prior probability distribution, *p*(θ)) with a likelihood (*p*(IC|θ)). Assuming Gaussian prediction errors, the log‐likelihood (log *p*(IC|θ)) is the negative sum of all squared prediction residuals (i.e., a larger error gives a smaller log‐likelihood value). Here, θ is the unknown parameter value and IC the initial conditions, that is, the observations used in the model parameterization (IPG and E‐OBS). Each parameter was assigned a uniform prior based on values found in the literature (Appendix S1), and the range was explored using an adaptive Metropolis–Hasting algorithm (AMH) (Andrieu & Thoms, 2008; Haario, Saksman, & Tamminen, 2001). For each model, the AMH was run eight times using randomly selected starting points and up to 300,000 iterations (Appendix S1). For each iteration, new parameters are randomly generated and the likelihood function (*L*_*i*_) scores the models ability to predict the observed budburst given the climate data. The *L*_*i*_ is then compared to that of the previous iteration (*L*_*i*−1_), if larger the new parameters are kept, else a new set of parameters based on either the latest accepted set or the new parameters is created. When the new parameters yield a smaller *L*, they are accepted if the ratio *L*_1_/*L*_*i*−1_ is larger than a random number (a ε U[0,1]). By occasionally keeping parameter values that give a smaller *L*, the AMH algorithm reduces the risk of getting stuck in local optima, allowing it to better explore the possible parameter values. Before reaching the posterior distribution, we removed the burn‐in period and thinned each chain to reduce autocorrelation. The burn‐in period was assumed to consist of all samples until the first time at which the likelihood exceeds the mean likelihood over the last 10% of the chain. For each parameter, the lag at which autocorrelation in the chain was <0.2 was identified. The common lag for all parameters was taken as the largest of the parameter lags, giving autocorrelations <0.2. Each chain was then thinned by only keeping samples separated by the common lag. For each set of eight starting points, only chains with markedly higher LogL were kept, removing chains that fail to converge to reasonable parameter values. A nonparametric two‐sample Kolmogorov–Smirnov test (KS test) was used to assess if posteriors of individual chains originate from the same distribution as posteriors of pooled chains at 5% significance level, that is, assessing the importance of different initial values in the AMH and its ability to converge to similar parameter values regardless of starting point.

The budburst models were selected based on their complementarity and success of the parameterization constrained by IC IPG_All_ (i.e., chain convergence) to generate ensemble projections that included representatives of all different model classes (MC). GDD_DOY_ and SIG_DOY_ provided different weighting of the forcing temperatures, and GDD_1_ was found to be complementary to them, as it does not assume a photoperiod requirement that potentially can limit advancement in budburst timing. One photoperiod model, BC_DOY_, was included along with two models accounting for both chilling and forcing temperatures, ALT and SEQ. It was not possible to obtain sufficient convergence for SEQ when trying a full parameterization, so three of the parameters were set to commonly accepted values; the starting day for chilling accumulation (October 1), and the minimum and maximum temperature threshold for chilling accumulation (−3.4°C and 10.4°C; e.g., Hänninen, 1990). UNI (sharing features with ALT and SEQ) was not included in the final ensemble as it did not converge sufficiently within 300,000 iterations (only one chain converging per provenance, and only ten iterations remaining after thinning for the early German provenance (P121), which indicates a hard optimization problem or an overparameterized model).

### 2.4 Boundary conditions

For parameterization, the budburst models were forced with interpolated observed daily mean air temperature with a spatial resolution of 0.44° (E‐OBS vs. 10.0, 1950–2014) (Haylock et al., 2008). Trees of each provenance within the same IPG were assumed to be under the same climatic influence. The IPGs were located in separate climate grid cells, except IPG_27_ and IPG_28_. To account for local temperature conditions at the IPGs, the E‐OBS temperature was adjusted using the elevation difference between the IPG and the corresponding grid cell, using a global standard temperature lapse rate of 6.4°C/km (Olsson & Jönsson, 2015).

The budburst model projections focused on three time periods: (i) 1971–2000 was selected for comparison with the model runs using E‐OBS data. These model runs differ as the E‐OBS data allows for chronological comparisons with budburst observations, as opposed to the transient climate model simulations that capture the climate conditions but not the year‐by‐year variations. Furthermore, the climate model data were not adjusted for site‐specific elevation differences, as the purpose was to generate simulations representing the grid‐cell level, mapping the entire study region of northern Europe. (ii) The period 2011–2040 was selected to assess current climate and near future, a period little influenced by uncertainties associated with future greenhouse gas emissions (IPCC, 2013). (iii) The period 2051–2080 was selected to quantify uncertainties in plant phenological response in relation to a high emission scenario (RCP 8.5). With a rotation period of at least 50 years, this period is highly relevant to consider during regeneration of Norway spruce forest stands.

To address uncertainties related to climate model data, the phenology models were driven by five climate model datasets from EURO‐CORDEX representing RCP8.5 at a spatial resolution of 0.44° (Jacob et al., 2014). The subensemble consisted of data from one regional climate model (RCM) SMHI‐RCA4 with boundary conditions defined by five general climate models (GCM); CanESM2, CERFACS CNRM CM5, IPSL CM5A MR, NorESM1‐M, and GFDL‐GFDL ESM2M. The subensemble had been selected to represent the variation of a larger ensemble of eleven RCM‐GCM combinations (Wilcke & Bärring, 2016) based on the variation in daily mean temperature and degree days for the periods of 1971–2000 and 2069–2098 in eight European subregions corresponding to the PRUDENCE regions (Christensen & Christensen, 2007; Pulatov et al., 2016). All ensemble members had been bias corrected, using empirical quantile mapping with EURO4M as reference dataset (Wilcke, Mendlik, & Gobiet, 2013). We hereafter refer to the BC datasets as CanESM2, CERFACS, IPSL, NorESM1, and GFDL.

### 2.5 Analysis

An ensemble was produced for each Norway spruce provenance (P121, P122, and P123) and time periods (TP1: 1971–2000, TP2: 2011–2040, and TP3: 2051–2080) by making multiple simulations over sets of six initial conditions (IC: IPG_All_, IPG_2_, IPG_7_, IPG_14_, IPG_33,_ and IPG_56_), seven budburst model (MC: MT, GDD_1_, GDD_DOY_, SIG_DOY_, BC_DOY_, ALT, and SEQ), a set of model parameter values, and five boundary conditions (BC: CanESM2, CERFACS, IPSL, NorESM1, and GFDL; Figure 1a). The predictive distribution, given observations and boundary conditions (*p*(MC|IC,BC)), can be seen as a collection of projections using different parameters (*k*), that is, *p*(MC|IC,BC) = sum_*k*_ *p*(MC|θ_*k*_,BC)*p(θ_*k*_|IC). The difference, or uncertainty, in the parameters is described by the joint posteriors represented in the thinned Markov chains. The joint posterior was sampled n*(100/M)+1 times for each of the M distinct local modes and *n* parameters in each model; the posterior sampling includes one sample of each local mode (the +1) and results in n*100 + M samples for each model.

The observed budburst and budburst projections based on climate model data were aggregated into different cumulative distribution functions (CDF) and compared using a nonparametric Kruskal–Wallis test (KW test) to test for equality among the distributions. The KW test statistic is the mean difference on sum of ranks, representing the area between CDFs (Figure 1b). CDF mean values were calculated and compared for quantitative assessments of changes between time periods. For multiple comparisons, we used Bonferroni‐corrected *p*‐values in a post hoc test to evaluate specific sample pairs for stochastic ordering at 5% significance level. To account for differences in sample size when comparing and plotting test statistics from multiple tests, the KW‐value was standardized through division with the width of their 95% confidence interval (CI). To obtain results on the provenance‐specific response to climate change, no aggregation was performed across different provenances or time periods.

The analysis included three parts. First, we benchmarked differences in observed budburst among provenances and IPGs during 1968–2013, using the KW test with Bonferroni‐corrected *p*‐values to evaluate if the individual time series originate from the same distribution. Thereafter, we estimated the overall model calibration accuracy for the time period 1971–2000, using adjusted coefficient of determination (adj. *R* ^2^) and Akaike's information criterion (corrected for sample size, AICc), between observed and E‐OBS‐simulated budburst. In order to keep the comparison across model parameterizations constant, the entire observational dataset was used for evaluation of all model varieties, that is, this step included external validation data for single‐site calibrations. Spatial differences in model accuracy were further evaluated using the KW test on the observed and predictive distributions resulting from model runs using climate model data.

Second, we used the ensemble mean to estimate the climate change impact on the timing of budburst, and the 25th and 75th percentile as a conservative measure of uncertainties. Potential changes in the spatial variation were evaluated using a general linear model (GLM) with annual ensemble mean across IPGs as independent variable and annual standard deviation as dependent variable (e.g., Menzel, Sparks, Estrella, & Roy, 2006). Changes in climate and budburst across the main area of Norway spruce distribution in Europe (north of the Alps) were projected using all BC, and included initial conditions IPG_All_, model classes MT, GDD_1_, GDD_DOY_, SIG_DOY_, BC_DOY_, and ALT, and boundary conditions CanESM2, CERFACS, IPSL, NorESM1, and GFDL (see Section 3.1).

Third, we assessed the sources of uncertainty in the projections by comparing projections aggregated into IC, MC, and BC, using the KW test with Bonferroni‐corrected *p*‐values. Differences among model classes were further evaluated with respect to the simulated response to a warmer climate, spatial differences, and sensitivity to initial conditions. All simulations and analysis were performed using the computational program Matlab 2015b. Two supplements provide additional information on model parameterization (Appendix S2) and provenance‐specific analysis (Appendix S2), referred to in the text as Table A1–A4, Fig. A1–A2 and Fig. B1–B6, respectively.

## 3 RESULTS

### 3.1 Observed budburst and model accuracy

The timing of budburst in 1968–2013 was approximately 1 week earlier for the early German provenance (P121) than for the late German (P122, Δ 6.48 ± 6.37 days) and late Norwegian (P123, Δ 7.54 ± 7.79 days) provenances (KW test, α = 0.05, *df* = 2). There was a significant variation among all IPGs (KW test, α = 0.05, *df* = 22); however, the five IPGs included to generate initial conditions (IC) were clustered in two groups: The timing of budburst did not differ significantly among IPG_2_, IPG_7,_ and IPG_33_, or between IPG_14_ and IPG_56_, for any of the provenances. The spatial variation in timing of budburst among all IPGs (measured as the annual standard deviation) did not differ significantly between years.

All combinations of models and calibration dataset (driven by site‐adjusted E‐OBS data) were able to reproduce observed timing of budburst (*p* < .001), although the IPG‐specific initial conditions influenced the ability of the phenology models to capture the timing of observed budburst (Table 3). For most models, the parameterization based on IPG_All_ generated among the highest degrees of explanation, but for all models, some parameterizations based on individual sites were as good. The model parameterizations with highest accuracy and best trade‐off between goodness of fit and model complexity differed somewhat among the three provenances: for P121 this was generated by GDD_DOY_ and IPG_33_ (*R* ^2^ = 0.68, AICc = 4234), for P122 by ALT and IPG_33_ (*R* ^2^ = 0.72, AICc = 3,992), and for P123 by BC_DOY_ and IPG_All_ (*R* ^2^ = 0.73, AICc = 3,965). GDD_1,_ GDD_DOY,_ and BC_DOY_ were the three models not failing to predict budburst for any site and year, and BC_DOY_ had generally a somewhat higher accuracy than GDD_DOY._ The RMSE of the different model versions varied between 6.9 and 13.6, in general being slightly lower for model calibrations with IPG_All_ (average RMSE 8) than for the single sites (average RMSE 9; Table 3). For all model calibrated with IPG_All,_ the bias was 0. Bias for single‐site calibrations evaluated using the entire dataset, varied between −8.2 for calibrations with IPG_14_ (the westernmost site with an oceanic climate) and +8.8 for calibrations with IPG_7_ (the northeastern site with a continental climate). The bias of IPG_2_ was −4.9, IPG_33_ 0.2, and IPG_56_ 1.7, generating an ensemble bias of 0.

**Table 3:** Model calibration accuracy (adj. *R* ^2^) and RMSE across International Phenological Gardens, for E‐OBS simulations evaluated with observed budburst in 1971–2000, for each of the initial conditions (IC: IPG_All_, IPG_2_, IPG_7_, IPG_14_, IPG_33,_ and IPG_56_) and three provenances (on early [P121] and two late [P122, P123])

Provenance	Model	Model accuracy						Model RMSE					
		IPG_All_	IPG_2_	IPG_7_	IPG_14_	IPG_33_	IPG_56_	IPG_All_	IPG_2_	IPG_7_	IPG_14_	IPG_33_	IPG_56_
P121	MT	0.57	0.57	0.55	0.57	0.57	0.57	9.2	9.2	9.4	9.2	9.2	9.2
	GDD_1_	0.65	0.63	0.51	0.61	0.57	0.59	8.3	8.6	9.9	8.8	9.3	9.0
	GDD_DOY_	0.65	0.66	0.66	0.68	**0.68**	0.65	8.3	8.2	8.2	8.0	8.0	8.4
	BC_DOY_	0.66	0.57	0.59	0.59	0.57	0.63	8.2	9.2	9.0	9.0	9.2	8.6
	SIG_DOY_	0.66	0.22	0.40	0.39	0.06	0.32	8.2	12.4	10.9	10.9	13.6	11.6
	ALT	0.62	0.64	0.61	0.53	0.67	0.47	8.6	8.4	8.8	9.6	8.1	10.3
	SEQ	0.63	0.36	0.40	0.35	0.52	0.50	8.6	11.3	10.9	11.3	9.8	10.0
P122	MT	0.55	0.55	0.55	0.55	0.55	0.55	8.6	8.6	8.7	8.6	8.6	8.7
	GDD_1_	0.70	0.69	0.54	0.65	0.62	0.65	7.0	7.1	8.7	7.6	7.9	7.6
	GDD_DOY_	0.68	0.71	0.69	0.71	0.71	0.70	7.2	6.9	7.2	6.9	6.9	7.0
	BC_DOY_	0.71	0.63	0.67	0.66	0.67	0.68	6.9	7.8	7.4	7.5	7.4	7.3
	SIG_DOY_	0.70	0.14	0.30	0.36	0.13	0.30	7.1	11.9	10.7	10.3	12.0	10.8
	ALT	0.70	0.67	0.66	0.63	**0.72**	0.48	7.0	7.4	7.5	7.8	6.8	9.3
	SEQ	0.68	0.29	0.41	0.34	0.51	0.29	7.3	10.8	9.9	10.4	9.0	10.9
P123	MT	0.54	0.54	0.52	0.54	0.54	0.54	9.1	9.1	9.3	9.1	9.1	9.1
	GDD_1_	0.70	0.72	0.54	0.65	0.63	0.55	7.3	7.1	9.0	7.9	8.2	8.9
	GDD_DOY_	0.66	0.71	0.65	0.69	0.69	0.68	7.8	7.2	7.9	7.4	7.4	7.6
	BC_DOY_	0.69	0.66	0.67	0.67	0.66	0.63	7.4	7.8	7.6	7.6	7.8	8.1
	SIG_DOY_	0.67	0.68	0.24	0.21	0.15	0.29	7.6	7.5	11.6	11.9	12.3	11.3
	ALT	**0.73**	0.71	0.67	0.52	0.73	0.45	6.9	7.1	7.7	9.2	6.9	9.9
	SEQ	0.64	0.29	0.40	0.30	0.50	0.26	8.0	11.2	10.3	11.2	9.5	11.5

All results are significant at the level of *p* < .001. Gray shading highlights the IC with highest model accuracy, and bold indicates the provenance‐specific models with lowest AICc (Akaike's information criterion corrected for sample size).

### 3.2 Budburst projections

#### 3.2.1 Model performance and failure

In general, the models failed to simulate budburst more often when constrained by initial conditions based on one IPG, compared to IPG_All_ (Table 4). MT and ALT failed more frequently in 1971‐2000, predominantly at the most northern site (IPG_7_) with IC IPG_2_ and IPG_14_. SEQ and SIG_DOY_ failed more frequently in the warmer climate of TP2 and TP3, but for two different reasons. SEQ failed for all provenances, boundary conditions, and initial conditions (predominantly with IC IPG_2_, IPG_14_ and IPG_33_) due to insufficient chilling accumulation. SIG_DOY_ failed for all provenances with IC IPG_2_, IPG_7_, IPG_14,_ and IPG_33_, which can be attributed to the overall uncertainty in the parameterization of growth rate (*b*) and inflection point (*c*) of the logistic function that determines the response rate to forcing temperatures. The two parameters are highly interdependent (Spearman's rank correlation *r*_*s*_ < −0.84, *p* < .001) with distributions containing both positive and negative values (Fig. A2). For simulations with a positive growth rate, irrespective of the sign of the inflection point, the response rate is lower with higher temperature.

**Table 4:** Model failure to simulate budburst before August 1, indicated as the percent of failure (max 100.00%) for each provenance (one early [P121] and two late [P122, P123]), model class, initial conditions, and time period (TP1: 1971–2000, TP2: 2011–2040, and TP3: 2051–2080)

Model	IPG_All_			IPG_2_			IPG_7_			IPG_14_			IPG_33_			IPG_56_		
	TP1	TP2	TP3	TP1	TP2	TP3	TP1	TP2	TP3	TP1	TP2	TP3	TP1	TP2	TP3	TP1	TP2	TP3
P121																		
MT	0	0	0	0.01	0	0	0	0	0	0.01	0	0	0	0	0	0	0	0
GDD_1_	0	0	0	0	0	0	0	0	0	0	0	0	0	0	0	0	0	0
GDD_DOY_	0	0	0	0	0	0	0	0	0	0	0	0	0	0	0	0	0	0
BC_DOY_	0	0	0	0	0	0	0	0	0	0	0	0	0	0	0	0	0	0
SIG_DOY_	0	0	0	0.09	0.18	0.44	0.01	0.02	0.11	0.01	0	0	0.14	0.31	0.75	0	0	0
ALT	0	0	0	0	0	0	0	0	0	0	0	0	0	0	0	0	0	0
SEQ	0.42	0.46	1.11	3.28	4.17	8.13	0.04	0.04	0.24	1.35	1.28	1.46	2.14	1.6	4.79	0.44	0.34	0.48
P122																		
MT	0	0	0	0.02	0	0	0	0	0	0.08	0.02	0	0.01	0	0	0	0	0
GDD_1_	0	0	0	0	0	0	0	0	0	0	0	0	0	0	0	0	0	0
GDD_DOY_	0	0	0	0	0	0	0	0	0	0	0	0	0	0	0	0	0	0
BC_DOY_	0	0	0	0	0	0	0	0	0	0	0	0	0	0	0	0	0	0
SIG_DOY_	0	0	0	0.08	0.15	0.37	0.13	0.06	0.28	0.05	0.1	0.38	0.95	0.33	0.9	0	0	0
ALT	0	0	0	0	0	0	0	0	0	0.15	0.07	0.03	0	0	0	0	0	0
SEQ	0.20	0.15	0.49	0.56	0.39	0.55	0.04	0.04	0.24	1.29	1.16	1.23	4.09	1.77	5.21	0.01	0	0.08
P123																		
MT	0	0	0	0.07	0.01	0	0	0	0	0.98	0.65	0.12	0.01	0	0	0	0	0
GDD_1_	0	0	0	0	0	0	0	0	0	0	0	0	0	0	0	0	0	0
GDD_DOY_	0	0	0	0	0	0	0	0	0	0	0	0	0	0	0	0	0	0
BC_DOY_	0	0	0	0	0	0	0	0	0	0	0	0	0	0	0	0	0	0
SIG_DOY_	0	0	0	0	0	0	0.06	0.15	0.44	0.13	0.33	0.92	0.15	0.36	1.08	0	0	0
ALT	0	0	0	0	0	0	0	0	0	0	0	0	0	0	0	0	0	0
SEQ	0.02	0.21	1.34	1.25	1.35	2.42	0.02	0.06	0.27	2.36	2.39	2.99	0.82	1.58	4.7	0.01	0	0.05

**Figure 2:**
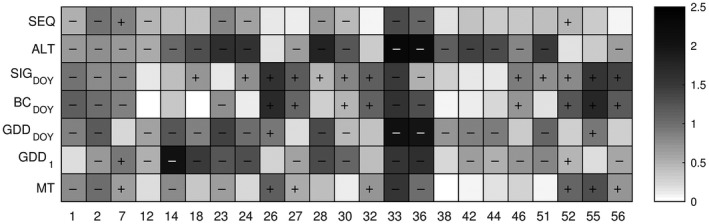
Site‐specific model accuracy for the early German provenance (P121), evaluating the similarity between distributions of budburst observations and simulations for 1971–2000 based on climate model data, aggregated for each combination of model class (along *y*‐axis) and International Phenological Garden (along *x*‐axis). The grayscale displays absolute values of standardized average differences between sum of ranks of observations and simulations (Kruskal–Wallis test, α = 0.05, *df* = 1). Significant differences indicate that the predictive distribution (on average) includes earlier (“−”) or later (“+”) budburst than the observed distribution. (See Appendix S2 for corresponding figures for provenance P122 and P123.)

The TP1 simulations, based on climate model data, were for some sites significantly earlier or later than observed budburst (KW test, α = 0.05, *df* = 1) (Figure 2). For some sites (i.e., IPG_2_, IPG_12_, IPG_23_, IPG_33,_ and IPG_36_), all models generated budburst projections that were early in comparison with observed timing. This is related to differences between E‐OBS data (adjusted for site‐specific altitude, used for model parameterization) and gridded climate model data (used for large‐scale and long‐term climate impact assessments). In comparison with observed timing of budburst, the models generated different grid‐cell‐specific projections for TP1, with ALT and SIG_DOY_ representing the two extremes: The ALT model projected an earlier than observed budburst for 19 of 23 grid cells with IPGs, whereas SIG_DOY_ projected an earlier than observed budburst for five grid cells, and later than observed for 12 of the grid cells.

#### 3.2.2 Ensemble projections

**Figure 3:**
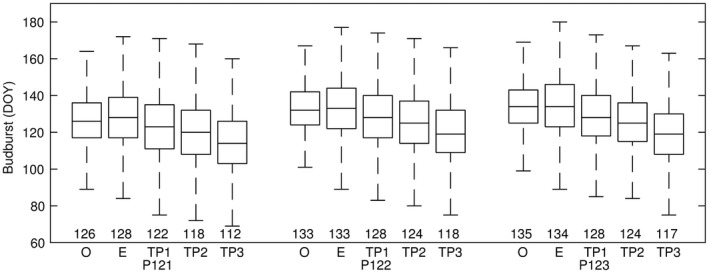
Box plots showing the ensemble mean (value), median (line), the 25th and 75th percentile (box) and 99.3% (whiskers) of day of year observed budburst (O), simulations forced by the (adjusted) interpolated observed temperature dataset E‐OBS (E), and simulations forced by modeled climate data (boundary conditions) across all 23 International Phenological Gardens. Observations and simulations were aggregated after provenance; one early (P121), and two late (P122 and P123), and time period; 1971–2000 (O, E and TP1), 2011–2040 (TP2), and 2051–2080 (TP3)

**Figure 4:**
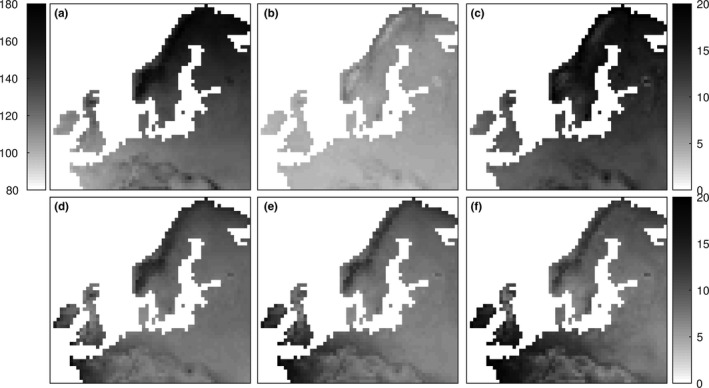
Spatial variation in budburst simulations for the early Germany provenance (P121). (a) Grid‐cell ensemble means (day of year) in 1971‐2000, (b) average change (number of days) from 1971–2000 to 2011–2040, and (c) average change (number of days) from 1971–2000 to 2051–2080. The lower panels show the standard deviations for each time period, (d) 1971–2000, e) 2011–2040, and (f) 2051–2080. The grid‐cell ensemble means are based on the initial conditions IPG_A__ll_, model classes MT, GDD_1_, GDD_DOY_, BC_DOY_, SIG_DOY_, and ALT, and boundary conditions CanESM2, CERFACS, IPSL, NorESM1, and GFDL (see Section 3.1 for subset selection). (See Appendix S2 for corresponding figures for provenance P122 and P123.)

The ensemble projections indicate that the temporal and spatial changes in budburst will be similar among the provenances, maintaining the interprovenance relationship over time, also in a warmer climate (Figure 3). The observed timing of budburst and simulations based on E‐OBS in 1971‐2000 agree to a large extent, but the range was slightly larger for the E‐OBS simulations, which reflects both model uncertainties, uncertainties associated with the E‐OBS data, and added variability by site‐year projections without corresponding budburst observations (i.e., gaps in the observed datasets). The discrepancies between model projections based on E‐OBS and modeled climate data were solely due to differences in the temperature estimates (see Section 2.4). For many of the sites, the climate model data indicated slightly warmer conditions that the E‐OBS data, as the climate model data were not adjusted for site‐specific altitudes. Sixteen of 23 sites were at lower altitudes than the corresponding grid‐cell average altitude (Table 1). That is, the E‐OBS data were adjusted to generate time series that represented the site‐specific conditions, for optimal calibration of the phenology models, but the climate model data were not adjusted to provide future predictions that represented the grid‐cell level, enabling mapping of the entire study region (c.f. Figure 4).

A subset of projections (initial condition IPG_All_, model classes MT, GDD_1_, GDD_DOY_, SIG_DOY_, BC_DOY,_ and ALT, and all boundary conditions) was combined to assess variations in climate sensitivity within the study region (Figure 4, Fig. B2). The budburst advancement was more pronounced in northern Europe, that is, in areas with a more pronounced warming during winter and spring, while the estimated interannual variation in budburst (as influenced by MC and BC) increased more in western Europe, that is, in areas where the current climate is relatively warm (Fig. B3). It is worth noting that even though the forcing requirement in GDD_1_ and GDD_DOY_ is highly correlated, GDD_1_ projects a greater advancement by accounting for temperature increase in late winter and early spring (i.e., before the starting day of GDD_DOY_, ranging from late February to late March, Table A2–A4).

#### 3.2.3 Model‐specific projections

**Figure 5:**
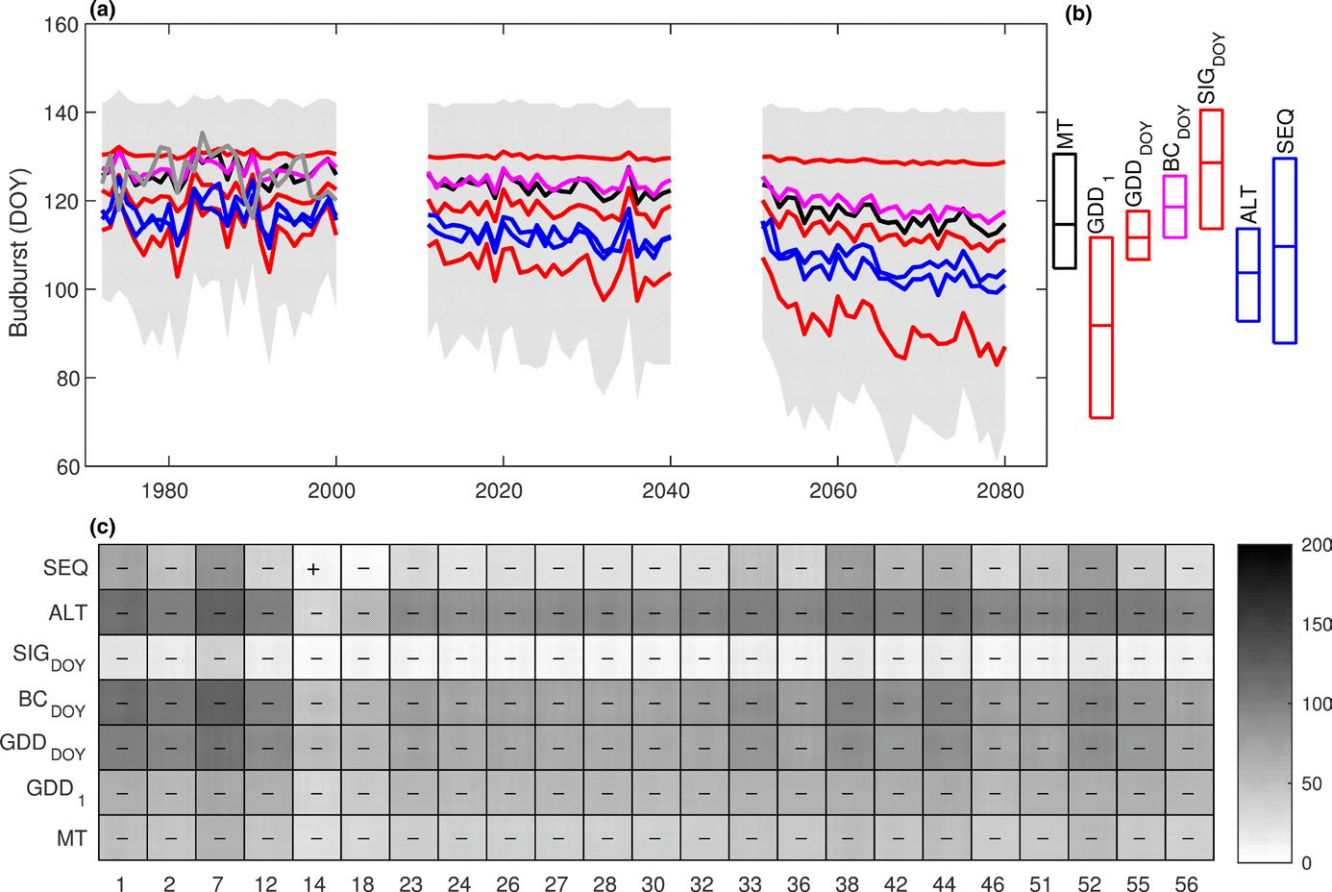
Three panels displaying the results of phenology model projections for the early provenance from Germany (P121) in relation to (a) interannual variation across the study region for the three selected time periods, (b) variation among phenology models for the last time period, and (c) variations in the strength of the climate change signal among models and International Phenological Gardens. The color in (a) and (b) group the models in relation to the physiological processes included the following: empirical (black), forcing (red), forcing modified by photoperiod (magenta), and chilling and forcing (blue), with the line representing the mean and the box the 25th and 75th percentile. In (c) the color displays absolute values of standardized average differences between sum of ranks of distributions aggregated for each combination of model class (along *y*‐axis) and International Phenological Garden (along *x*‐axis) (Kruskal–Wallis test, α = 0.05, *df* = 1). Significant differences (negative “−” or positive “+”) in simulations between 1971–2000 and 2051–2080 (Kruskal–Wallis test, α = 0.05, *df* = 1). (See Appendix S2 for corresponding figures for provenance P122 and P123.)

A comparison among the climate change projections generated by the different phenology models showed that GDD_1_ gave the strongest climate change signal, in terms of earlier budburst, followed by ALT, SEQ, GDD_DOY_, MT, BC_DOY,_ and SIG_DOY_ (Figure 5a). For each provenance and time period, the projected interannual variability (standard deviation of annual means) was highest with GDD_1_ and lowest with SIG_DOY_, and most models projected higher interannual variability in 2051–2080 (Figure 5b) than in 1971–2000. SIG_DOY_ and SEQ deviated from this pattern, as the temperature increase leads to increased model failure (see Section 3.2.1) and thereby an apparent decrease in variability.

SEQ indicated a larger spatial variation in projected budburst advancement from 1971–2000 to 2051–2080 than the other models (Figure 5c). Furthermore, SEQ was the only model to project a relative delay in budburst, at IPG_14_ for all provenances and at IPG_18_ for the two late provenances (P122 and P123, Fig. B4). Among all IPG sites, these two have the highest average temperature and lowest variability in winter (November–January) and spring (February–April). All temperature sum models projected the greatest advancement at the most northern site (IPG_7_), and the least advancement (or even a delay according to SEQ) at the most western site (IPG_14_). Minor differences were found for IPG_27_ and IPG_28_, even though they belong to the same climate grid cell, due to the latitude‐specific day length function implemented in BC_DOY_.

### 3.3 Sources of uncertainty

**Figure 6:**
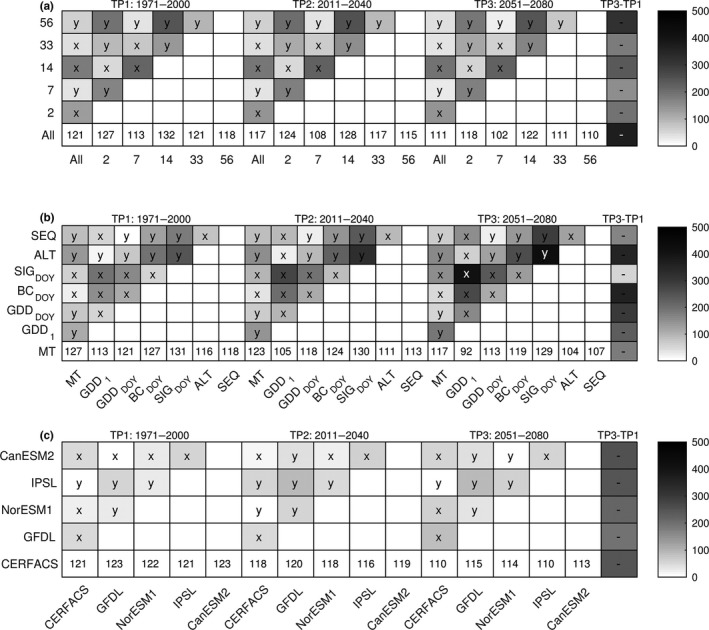
The relative contribution of initial conditions (IC), model classes (MC), and boundary conditions (BC) to uncertainties in the model projections for each time period. Differences in budburst simulations for the early provenance from Germany (P121) among cumulative density functions across (a) IC, (b) MC, and (c) BC. The distribution averages are presented along the *x*‐axis. The grayscale displays absolute values of standardized average differences between sum of ranks of the distributions (Kruskal–Wallis test with Bonferroni‐corrected *p*‐values, α = 0.05, (a) *df* = 6, (b) *df* = 6, and (c) *df* = 5). Significant differences in the post hoc pairwise comparison indicate the distribution that (on average) includes earlier budburst (*x*‐axis “*x*” or *y*‐axis “*y*”). The far right panels indicate significant differences (negative “−” or positive “+”) in simulations between 1971–2000 and 2051–2080 for the distribution on the *y*‐axis (Kruskal–Wallis test, α = 0.05, *df* = 1). (See Appendix S2 for corresponding figures for provenance P122 and P123.)

Four categories of model settings (provenance, IC, MC, and BC) were assessed for each time period by comparing projections across IPGs aggregated into different sets of cumulative distribution functions (CDFs, Figure 1b). All CDFs indicated an earlier timing of budburst in response to a warmer climate (Figure 6). The estimated average advancement in TP2 was 4.1 ± 1.6 days, and in TP3 10.2 ± 3.7 days. All provenances indicated the same magnitude of change, but the CDFs of the early provenance differed somewhat from the two late provenances, with a slightly larger variation within IC (11 days) than MC (8 days) and BC (6 days; c.f. Figure 6 and Fig. B5).

In this study, the variation was mainly attributed to the budburst models (MC), followed by the initial conditions (IC) and selection of climate model data (BC) (Figure 6). Within each category, the variability among CDFs increased over time, somewhat more for MC than for IC (Figure 6, Fig. B5). Among the initial conditions (IC‐CDFs), IPG_All_ changed the most and IPG_7_ the least. Among the phenology models (MC‐CDFs), BC_DOY_ showed the largest change and SIG_DOY_ the smallest change. A comparison of the climate change effect on the central tendency (i.e., change in average values between TP1 and TP3) revealed minor differences among IC‐CDFs and BC‐CDFs (all within the range of 8–11 days). Larger variations were found among the MC‐CDFs, ranging between 2 days (SIG_DOY_) and 22 days (GDD_1_; Table 5). The weak signal by SIG_DOY_ can be attributed to the increased frequency of model failure, and that the model in general is approaching failure (Table 4). Furthermore, the interannual variations predicted by BC_DOY_ were substantially smaller than for GDD_1_ (c.f. Figure 5b). Nevertheless, BC_DOY_ differed significantly between TP1 and TP3, indicating a general shift in timing of budburst by 9 days (i.e., close to the ensemble average).

**Table 5:** Differences between time periods (TP1 = 1971–2000, TP2 = 2011–2040, and TP3 = 2051–2080) among cumulative distribution functions (CDFs), comparing model projections grouped by initial conditions (IC), model classes (MC), and boundary conditions (BC)

Group	Average differences, TP2–TP1								Average differences, TP3–TP1							
IC	ENS‐AVG	All	IPG_2_	IPG_7_	IPG_14_	IPG_33_	IPG_56_		ENS‐AVG	All	IPG_2_	IPG_7_	IPG_14_	IPG_33_	IPG_56_	
P121	−3.8	−4	−3	−5	−4	−4	−3		−9.7	−10	−9	−11	−10	−10	−8	
P122	−4.0	−4	−4	−5	−4	−4	−3		−9.8	−11	−9	−11	−10	−10	−8	
P123	−4.0	−4	−4	−5	−4	−4	−3		−9.8	−11	−9	−11	−10	−10	−8	

MC		MT	GDD_1_	GDD_DOY_	BC_DOY_	SIG_DOY_	ALT	SEQ		MT	GDD_1_	GDD_DOY_	BC_DOY_	SIG_DOY_	ALT	SEQ
P121	−4.1	−4	−8	−3	−3	−1	−5	−5	−10.3	−10	−21	−8	−8	−2	−12	−11
P122	−4.1	−3	−9	−3	−4	0	−5	−5	−10.6	−10	−22	−9	−9	0	−13	−11
P123	−4.7	−5	−9	−4	−3	−1	−6	−5	−11.7	−13	−22	−10	−9	−3	−14	−11

BC		CERFACS	GFDL	NorESM1	IPSL	CanESM2				CERFACS	GFDL	NorESM1	IPSL	CanESM2		
P121	−3.8	−3	−3	−4	−5	−4			−9.6	−11	−8	−8	−11	−10		
P122	−4.2	−3	−3	−5	−5	−5			−9.8	−11	−9	−9	−10	−10		
P123	−4.2	−3	−3	−5	−5	−5			−10.4	−12	−9	−9	−11	−11		

Ensemble averages (ENS‐AVG) are presented, along with average differences for the individual ensemble members. Values are provided for three provenances, one early (P121) and two late (P122, P123).

A post hoc analysis, generating a pairwise comparison among IC‐CDFs and MC‐CDFs, showed that the ranking among model runs did generally not differ across time periods. However, the intricate interplay between parameter values and temperature generated some inconsistencies: The P123‐projections were generally earlier with IC IPG_56_ than with IC IPG_33_ in TP1, but later in TP2 and TP3 (Fig. B5), and the shift was induced by a stronger temperature response in IC‐IPG_33_ than IC‐IPG_56_, as indicated by the MT parameter values (IPG_33_: *k* = −5.5 and IPG_56_: *k* −3.2, Table A4). The projections with ALT were for all provenances generally earlier than with GDD_1_ in TP1, but later in TP3. With warmer springs, GDD_1_ will unconditionally project earlier budburst, while the advancement with ALT is partly offset by warmer winters, as fewer chilling days increase the forcing requirement of ALT. The BC‐CDFs showed minor differences in ranking among the time periods, as influenced by the partly random interannual variation in climate model data, and all BC‐CDFs indicated a similar magnitude of change between TP1 and TP3. The selection of initial conditions had a significant influence on all model projections (KW test, α = 0.05, *df* = 5), with SIG_DOY_ and SEQ being more sensitive than the other models (Fig. B6a,b).

## 4 DISCUSSION

Assessments of climate impacts on tree phenology are required for a range of theoretical and practical applications, including ecosystem modeling for mitigation and adaptation purposes and selection of suitable provenance at the timing of regeneration of forest stands (Migliavacca et al., 2012; Skrøppa & Steffenrem, 2016). A range of phenological models has been developed to capture temperature effects on the timing of budburst, differing in terms of model structure and complexity (Olsson & Jönsson, 2014). As there are remaining uncertainties on how to model the tree species and provenance‐specific phenology (Fu et al., 2015; Jochner, Sparks, Laube, & Menzel, 2016; Suvanto, Nöjd, Henttonen, Beuker, & Mäkinen, 2016), future projections should preferably be based on an ensemble of models with different structures (Basler, 2016). In this study, focusing on Norway spruce in northern Europe, we applied an ensemble approach using seven phenology models to provide a general overview of trends and uncertainties, addressing effects related to initial condition for model parameterization, model class, and boundary conditions. Our results are in line with other studies concluding that structurally different phenology models can generate similar results under current climate conditions, but differ in terms of future projections (Basler, 2016; Linkosalo, Lappalainen, & Hari, 2008; Vitasse et al., 2011). The ensemble projection indicated that the timing of budburst will be on average 10.2 ± 3.7 days earlier in 2051–2080 than in 1971–2000, given climate conditions corresponding to RCP 8.5. An earlier timing of budburst was associated with increased spatial variation, which is in line with observed changes for many species in Germany between 1951 and 2002 (Menzel et al., 2006). The variations captured by the ensemble projections were primarily caused by differences among phenology model classes (MC), secondly, by the initial conditions used for model parameterization (IC), and lastly, by climate model data (BC).

The ensemble analysis of this study focused on the complementarity of bud burst models, that is, the main source of variation. The included models captured different aspects of environmental regulation, such as forcing, chilling, and photoperiod, thereby contributing to the overall picture of current understanding of climate impacts on tree phenology (Fu, Campioli, Van Oijen, Deckmyn, & Janssens, 2012; Vitasse et al., 2011). Model parameterizations and projections were carried out for one early and two late Norway spruce provenances, as tree breeding with selection of suitable seed sources, (including provenances from e.g., Germany, Poland, and Belarus) is part of the climate adaptation strategy of the north European forestry sector. Tree phenology is an important selection criterion (Hannerz, 1999), as trees that start growth early in the season face a higher risk of frost damage, whereas trees that start late may not take advantage of the full growing season (Karlsson, 2009). Seed sources with late bud flushing are commonly recommended in south Sweden, whereas early bud flushing varieties are commonly used in north Sweden, as the spring temperature progression of this region is less frequently interrupted by frost episodes, and the growing season is shorter (Jönsson, Linderson, Stjernquist, Chlyter, & Bärring, 2004). The results of this study indicated that the temporal differences in timing of budburst between early and late provenances will remain in a warmer climate; however, the magnitude of temperature change will influence the regions and provenances most at risk. This is due to the fine balance between warmer climate generally reducing the number of frost days and tree dehardening and budburst occurring earlier in the year, at a time when temperature backlashes are more frequent (Jönsson & Bärring, 2011). While it was beyond the scope of this study to assess the risk of frost damage, it pointed toward the need of analyzing both the general trend in timing of budburst (most pronounced in northern Europe) and the interannual variation (will increase most in western Europe), as both aspects influence the risk.

The choice of observational data for model calibration was the second most influential factor. Calibrations based on IPG_All_ generated among the highest degrees of explanation for all models, although some of the single‐site calibrations, for which the calibration accuracy also included external validation data, generated as high *R* ^2^ values. This indicates some skill of the models to account for different climate conditions than calibrated for, although the single‐site calibration of SIG_DOY_, ALT, and SEQ performed generally less well than for GDD_1,_ GDD_DOY,_ and BC_DOY_. By minimizing the sum of residuals during parameterization, the budburst models become tuned around the average phenological temperature response of the initial conditions the models, although this does not compensate for lack of mechanistic understanding. The models tend to overestimate the temperature response when extrapolated to other conditions, with the implication that budburst is simulated too late in colder regions and too early in warmer regions (Olsson & Jönsson, 2014). The higher degree of model failure with initial conditions based on single IPGs indicated that the overestimation effect became more pronounced when the parameterization was constrained by local conditions than by average regional conditions (IPG_All_), especially when adding the effects of climate change.

In this study, UNI was not properly calibrated and thereby omitted from the ensemble projections. Previous studies have indicated that this model is prone to fit random noise data (Linkosalo et al., 2008), which results in poor performance at external sites (Fu et al., 2012), and this may have impaired our calibration process. Similar results have been found for SEQ (Fu et al., 2012), which in this study had the highest rate of failure to predict budburst, likely due to insufficient monitoring data for the calibration of chilling requirement (Fu et al., 2012; Linkosalo et al., 2008; Vitasse et al., 2011). The models that did not fail, GDD_1_, GDD_DOY,_ and BC_DOY_, were easiest to calibrate and among the better performing models when comparing model simulations with observed timing of budburst. Even so, the calibration procedure of these models highlights some model weaknesses and gaps in physiological understanding. The parameterization of GDD_1_ generated a posterior bimodality caused by the interdependence between base temperature and forcing requirement, and the parameterization of SIG_DOY_ was strongly influenced by the correlation between growth rate and infliction point. The calibrated exponential parameter of BC_DOY_ became close to one for all three provenances; thus, the forcing temperature accumulation was only marginally influenced by photoperiod. That is, while the calibrated interaction among starting day, temperature threshold and photoperiodic effect sufficiently capture current conditions, it may not address the dynamic between temperature and photoperiod in a warmer climate. This points to the general problem of estimating under which future climate conditions the model results are valid, further complicated by the fact that the climate development is scenario dependent (influenced by human actions) and the temperature increase will display latitudinal and seasonal variations (IPCC 2013). The result of a tree phenology study including a subselection of cold years, to enable a validation based on currently climate (Olsson & Jönsson, 2014), suggests that the model performance may deteriorate gradually with temperature increase as years with weather and climate conditions outside the model predictive capacity increase in frequency.

In this study, the boundary conditions were ranked as the third most important source of variation. However, the study design will influence the attribution of uncertainties (Uusitalo, Lehikoinen, Helle, & Myrberg, 2015) and it has to be taken into account that the model runs were based on bias‐corrected climate model data, selected to represent RCP 8.5 only (Wilcke & Bärring, 2016). In a study on Harvard forest (Massachusetts, USA), largest uncertainty was attributed to the selection of climate data, followed by budburst models and model parameterization (Migliavacca et al., 2012). The two scenarios included, A1fi and B1, have a median temperature increase by 2100 similar to RCP8.5 and RCP4.5 (Rogelj, Meinshausen, & Knutti, 2012), thereby capturing parts of the scenario uncertainty that was not specifically addressed in this study. Limited time and computer resources commonly restrict the possibility to take the entire range of climate model data into account when carrying out impact assessments (Wilcke & Bärring, 2016), and this study focused on a pronounced climate change scenario as this also captures aspects of what could happen in a less severe scenario. That is, the climate conditions outlined by RCP 8.5 for the midcentury correspond roughly with the conditions outlined by RCP 4.5 for the end of the century (IPCC, 2013).

## 5 CONCLUSION

In this study, we applied (i) Bayesian inference to estimate model parameter values to address uncertainties associated with selection of observational data, (ii) climate data selection to identify a subensemble of climate model data representative of a larger dataset, and (iii) ensembles modeling over multiple initial conditions, model classes, model parameterizations, and boundary conditions to generate future projections and uncertainty estimates. Structurally complex models were more likely to fail predicting budburst for some combinations of site and year than simple models, however, contributing to the overall picture of current understanding of climate impacts on tree phenology by capturing additional aspects of temperature response, for example, chilling. Model parameterizations based on single sites were more likely to result in model failure than parameterizations based on multiple sites, highlighting that the model parameterization is sensitive to initial conditions and may not perform well under other climate conditions, whether the change is due to a shift in space or over time. By addressing a range of uncertainties, this study showed that ensemble modeling provides more a robust impact assessment than would a single phenology model run.

## CONFLICT OF INTEREST

None declared.

## AUTHOR CONTRIBUTIONS

All authors contributed to all parts of the study. CO was involved in study design, modeling, and analysis; SO and JL contributed to the study design, recommended statistical methodology, and helped with interpretation of results, AMJ initiated the study, participated in analysis, and wrote the manuscript together with CO.
