# Supplementary material for: Trends and uncertainties in budburst projections of Norway spruce in Northern Europe
Source: Ecol Evol. 2017 Oct 22;7(23):9954–69. doi: 10.1002/ece3.3476 (PMC5723629; doi:10.1002/ece3.3476)

**Appendix S1**

Trends and uncertainties in projections of Norway spruce budburst in Northern Europe

Cecilia Olsson^1^*, Stefan Olin^1^, Johan Lindström^2^ and Anna Maria Jönsson^1^

^1^ Department of Physical Geography and Ecosystem Science, Lund University, SE-223 62 Lund

^2^ Centre for Mathematical Sciences, Lund University, SE-221 00 Lund

* Corresponding author: Anna Maria Jönsson

E-mail: Anna_Maria.Jonsson@nateko.lu.se

Tel.: +46-46-222 94 10

**Figure A1**: Map of selected IPG sites and visualisation of the P121 time series of budburst observations

**Table A1**: Model parameter definitions

**Table A2**: Model parameter values, P121

**Table A3**: Model parameter values, P122

**Table A4:** Model parameter values, P123

**Figure A2**: Successful and failed simulations


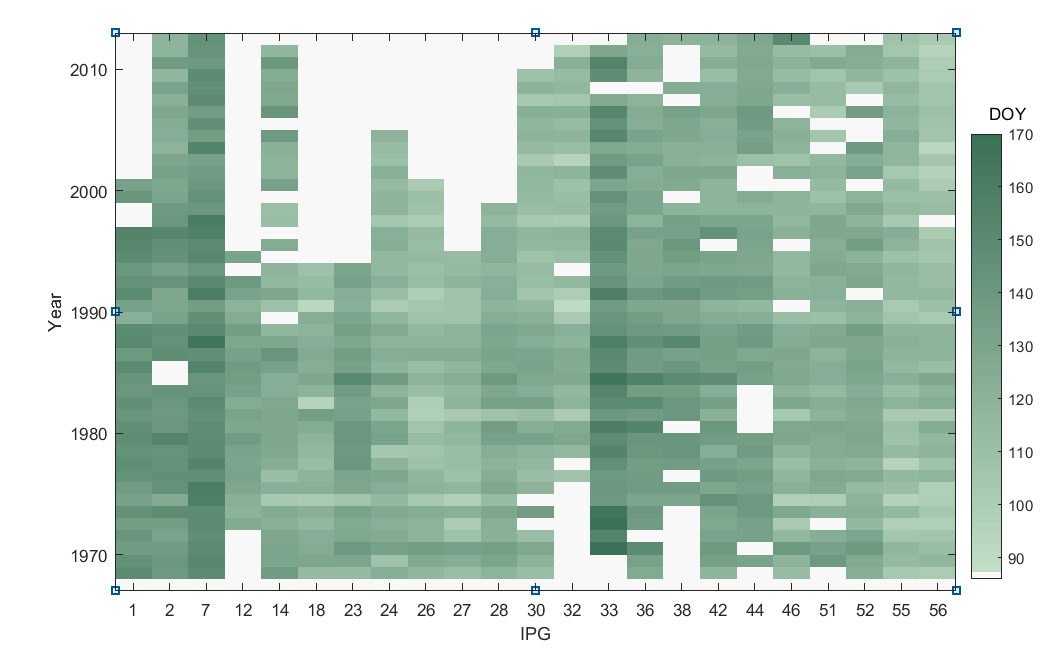

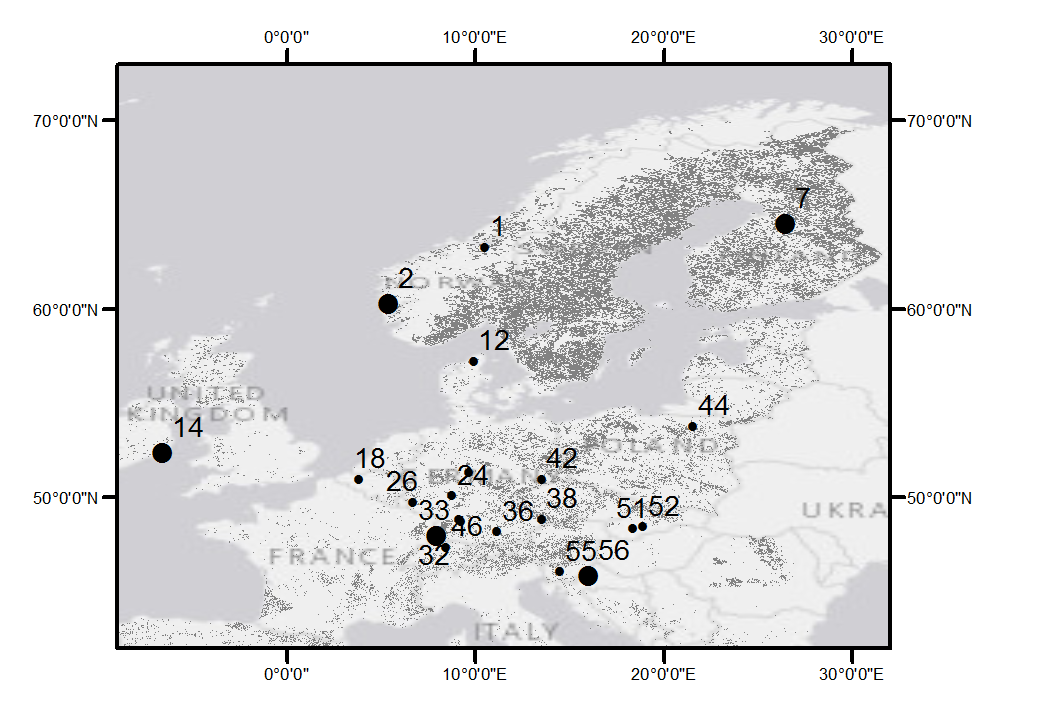


A1a)

A1b)

**Figure A1** a) Map over the 23 International Phenological Gardens included in this study, including the IPG number. The five larger dots display the single calibration sites. Grey shading over land area indicate regions with coniferous forest according to the CORINE land cover 2012 raster data, provided by EEA. b) Time series of budburst data, exemplified by provenance P121, for the 23 IPGs and the time period 1968 to 2013. The color scale indicate the day of year with budburst (DOY) for each site.

**Table A1** Model parameter definitions, with parameter range of the prior uniform distribution (p(θ)) and number of iterations used in the Bayesian inference (MHMC steps).

| **Model** | **Parameter** | **Definition** | **Range (p(θ))** | | **MHMC steps** |
| --- | --- | --- | --- | --- | --- |
|  | t | Day of the year |  |  |  |
|  | T_t_ | Temperature on day t (°C) |  |  |  |
|  | DL_t_ | Day length at day t (h) |  |  |  |
|  |  |  |  |  |  |
| GDD_1_ | T_b_ | Base temperature (°C) | -4 | 10 | 100,000 |
|  | F_crit_ | Forcing requirement (FU) | 10 | 2000 |  |
| GDD_DOY_ | t_0_ | Starting day for forcing accumulation (DOY) | 1 | 80 | 100,000 |
|  | T_b_ | Base temperature (°C) | -4 | 10 |  |
|  | F_crit_ | Forcing requirement (FU) | 10 | 2000 |  |
| SIG | T_b_ | Base temperature (°C) | -4 | 10 | 300,000 |
|  | b | Constant | -2 | 1 |  |
|  | c | Constant | -40 | 10 |  |
|  | F_crit_ | Forcing requirement (FU) | 10 | 2000 |  |
| SIG_1_ | b | Constant | -2 | 1 | 300,000 |
|  | c | Constant | -40 | 10 |  |
|  | F_crit_ | Forcing requirement (FU) | 10 | 2000 |  |
| SIG_DOY_ | t_0_ | Starting day for forcing accumulation (DOY) | 1 | 80 | 300,000 |
|  | b | Constant | -2 | 1 |  |
|  | c | Constant | -40 | 10 |  |
|  | F_crit_ | Forcing requirement (FU) | 10 | 2000 |  |
| BC_DOY_ | t_0_ | Starting day for forcing accumulation (DOY) | 1 | 80 | 100,000 |
|  | T_b_ | Base temperature (°C) | -4 | 10 |  |
|  | EXPO | Constant | 0.1 | 4 |  |
|  | F_crit_ | Forcing requirement (FU) | 10 | 2000 |  |
| ALT | T_b_ | Base temperature (°C) | -4 | 10 | 300,000 |
|  | t_1_ | Starting day for chilling accumulation (DOY) | 274 | 356 |  |
|  | β | Constant | 0.1 | 600 |  |
|  | γ | Constant | -1 | 1 |  |
| SEQ | T_opt_ | Optimum temperature for chilling (°C) | -10 | 15 | 300,000 |
|  | t_1_ | Starting day for chilling accumulation (DOY) | 274 | 356 |  |
|  | C_crit_ | Chilling requirement (CU) | 1 | 400 |  |
|  | T_b_ | Base temperature (°C) | -4 | 10 |  |
|  | b | Constant | -2 | 1 |  |
|  | c | Constant | -40 | 10 |  |
|  | F_crit_ | Forcing requirement (FU) | 1 | 2000 |  |
| UNI | aC | Constant | -4 | 4 | 300,000 |
|  | cC | Constant | -30 | 10 |  |
|  | bC | Constant | -25 | 25 |  |
|  | t_1_ | Starting day for chilling accumulation (DOY) | 274 | 356 |  |
|  | β | Constant | -50 | 600 |  |
|  | γ | Constant | -1 | 1 |  |
|  | C_crit_ | Chilling requirement (CU) | 1 | 400 |  |
|  | dF | Constant | -10 | 10 |  |
|  | fF | Constant | -40 | 10 |  |

**Table A2** Parameter values (mean, 25^th^ and 75^th^ percentiles) for the early German provenance (P121), from the predictive distributions obtained for different initial conditions (IC): IPG_All_, IPG_2_, IPG_7_, IPG_14_, IPG_33_ and IPG_56_.

**Table A3** Parameter values (mean, 25^th^ and 75^th^ percentiles) for the late German provenance (P122), from the predictive distributions obtained for different initial conditions (IC): IPG_All_, IPG_2_, IPG_7_, IPG_14_, IPG_33_ and IPG_56_.

**Table A4** Parameter values (mean, 25^th^ and 75^th^ percentiles) for the northern Norway provenance (P123), from the predictive distributions obtained for different initial conditions (IC): IPG_All_, IPG_2_, IPG_7_, IPG_14_, IPG_33_ and IPG_56_.

**Figure A2** Boxplots showing model parameter values, in relation to successful (black boxes) and failed projection (red boxes) for a range of sites (23 IPGs, Table 1) and years (three time periods of 30 years). Model failure was defined as budburst not simulated to occur before August 1, for at least one combination of site and year. The five sub-sections (A1a-e) correspond to one model each, with information for all model parameters (column title), parameter value (y-axis), time periods (x-axes: TP1 (1971-2000), TP2 (2011-2040) and TP3 (2051-2080)) and provenance (panel row: P121, (top panel), P122 (mid panel) and P123 (lower panel)). Sub-sections were included for the following models: a) MT, b) GDD_1_, c) SIG_DOY_, d) ALT and e) SEQ. GDD_DOY_ and BC_DOY_ were not included as they were without failed simulations. The boxplots highlight the median (line), the 25th and 75th percentile (box) and 99.3% (whiskers) of parameter values of the predictive distribution).


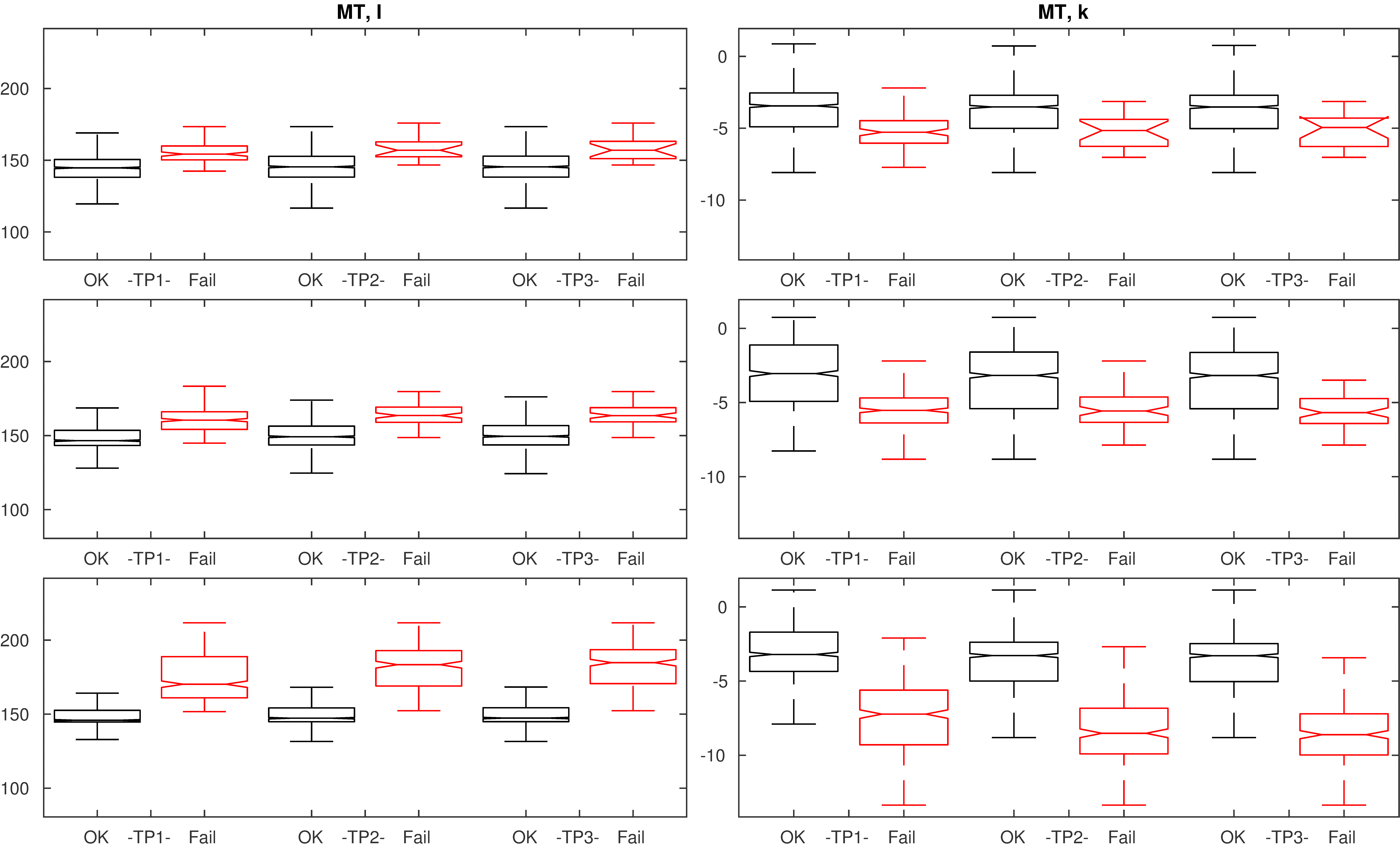


A2a)


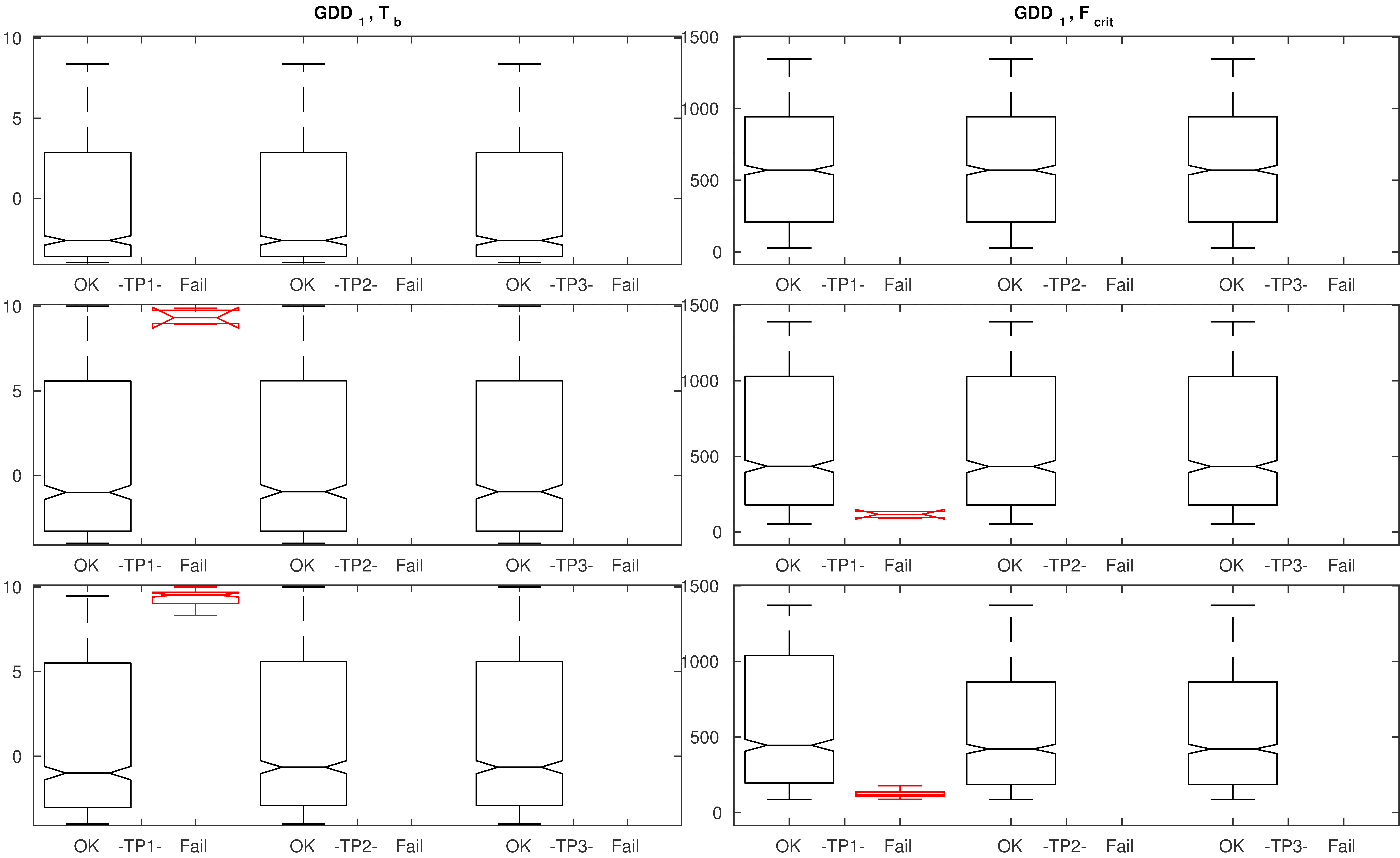


A2b)

A2c)


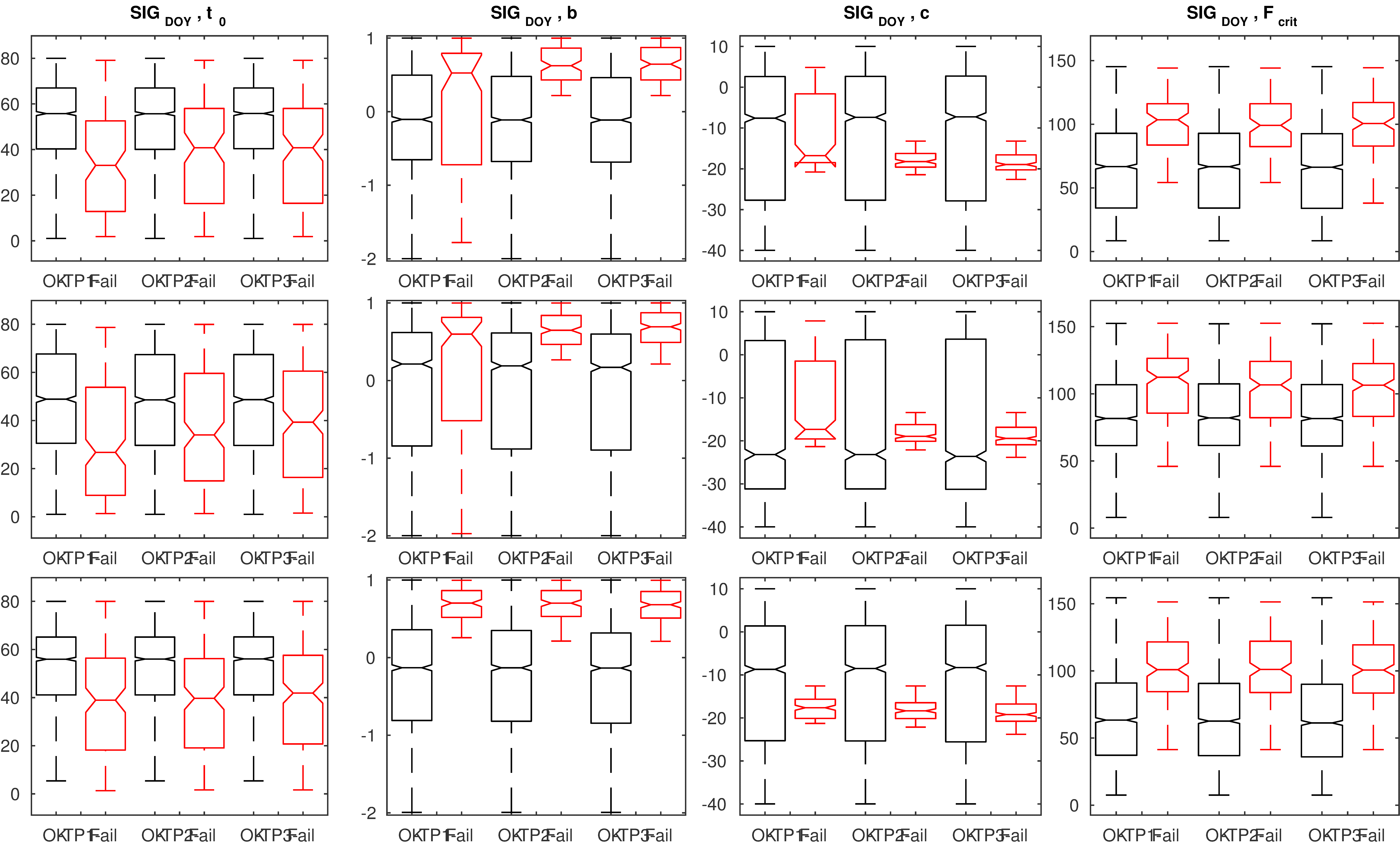


A2d)


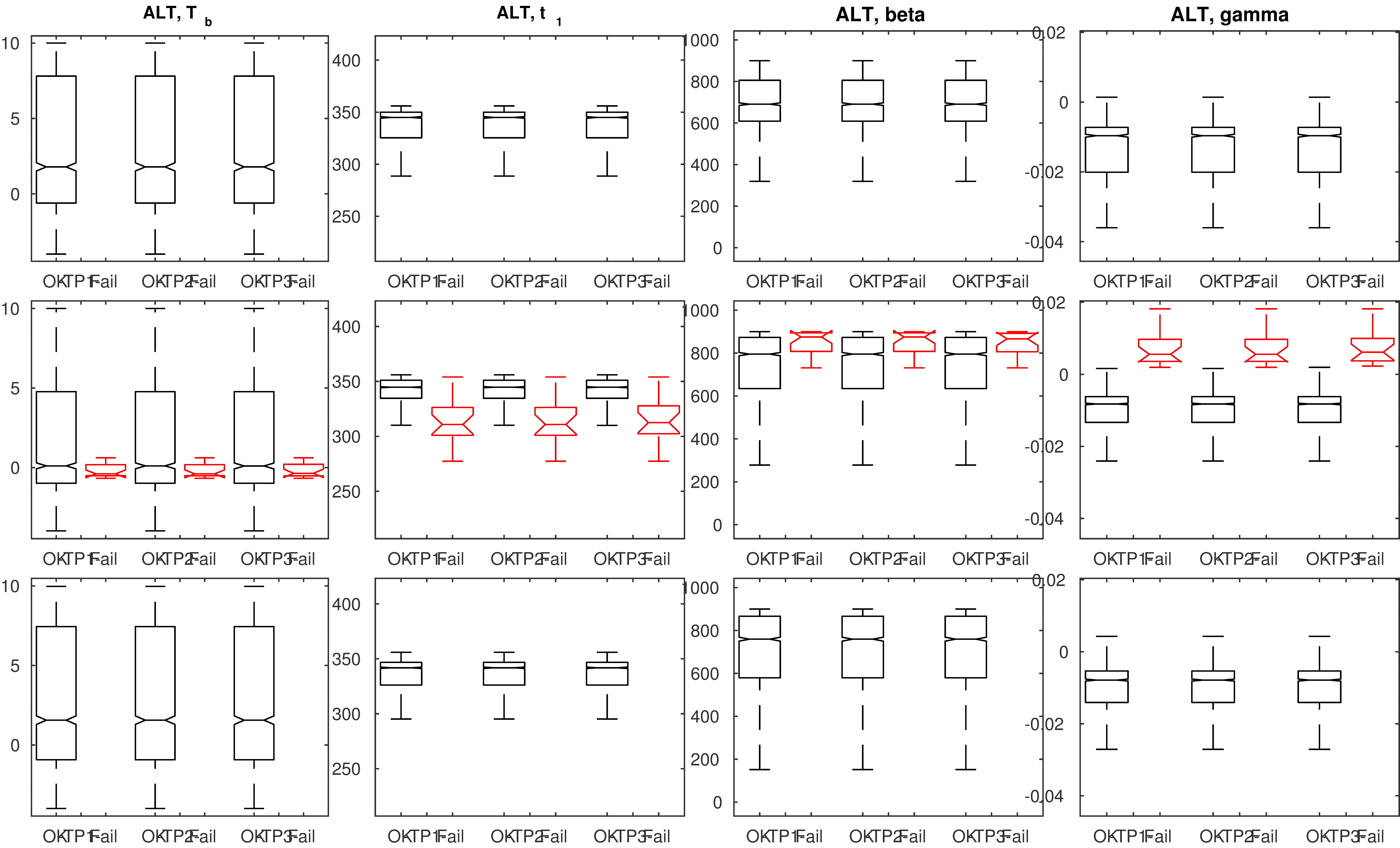


A2e)


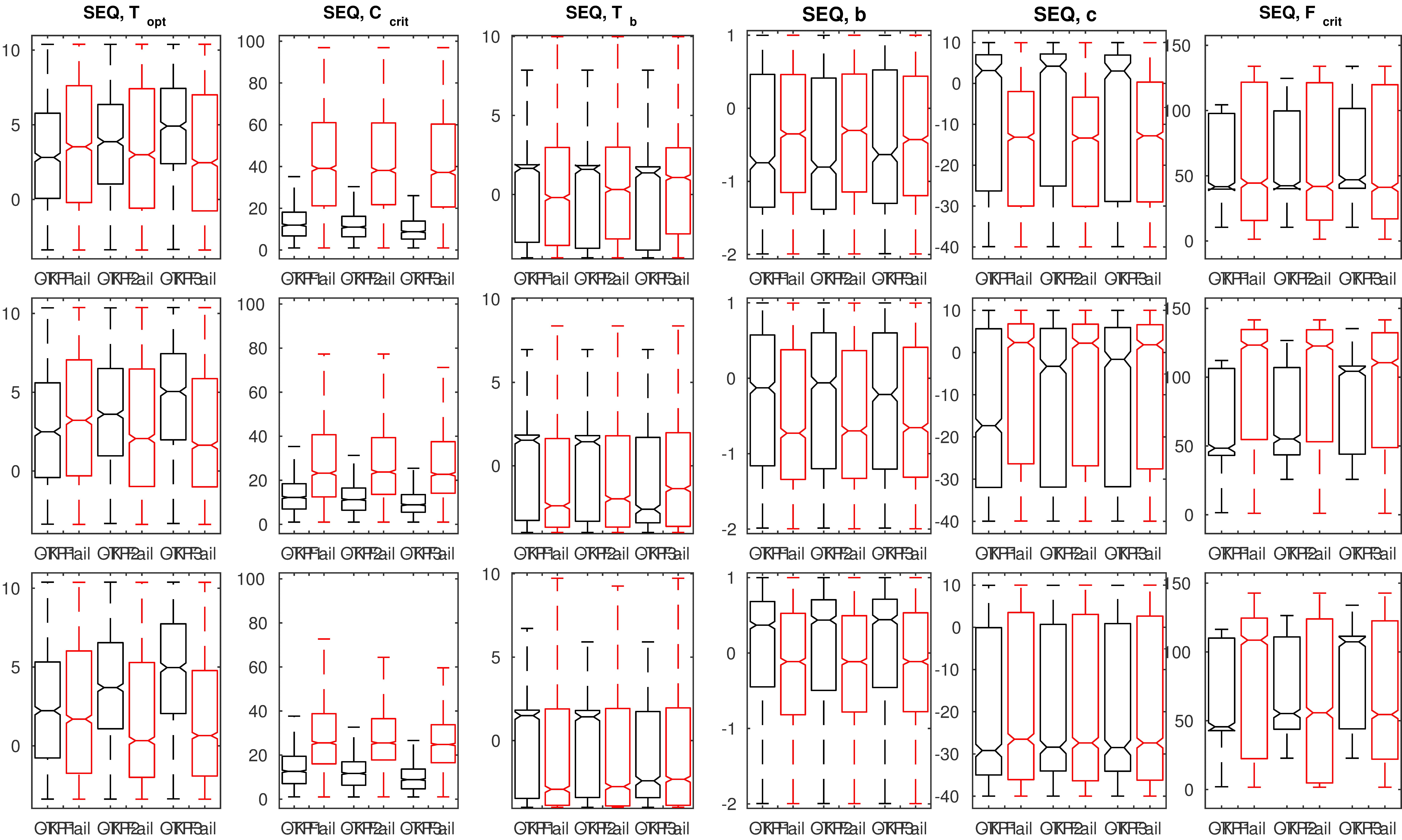

Supplement: Supplementary file 1 [file ECE3-7-9954-s001.doc]
