# Supplementary material for: Trends and uncertainties in budburst projections of Norway spruce in Northern Europe
Source: Ecol Evol. 2017 Oct 22;7(23):9954–69. doi: 10.1002/ece3.3476 (PMC5723629; doi:10.1002/ece3.3476)
Supplement: Supplementary file 2 [file ECE3-7-9954-s002.doc]

**Appendix S2**

Trends and uncertainties in projections of Norway spruce budburst in Northern Europe

Cecilia Olsson^1^*, Stefan Olin^1^, Johan Lindström^2^ and Anna Maria Jönsson^1^

^1^ Department of Physical Geography and Ecosystem Science, Lund University, SE-223 62 Lund

^2^ Centre for Mathematical Sciences, Lund University, SE-221 00 Lund

* Corresponding author: Anna Maria Jönsson

E-mail: Anna_Maria.Jonsson@nateko.lu.se

Tel.: +46-46-222 94 10

Figures of the ensemble mean temperature (B3), and figures (B1-B2, B4-B5) representing provenance P122 (late timing of budburst, originating from Germany) and P123 (late timing of budburst, originating from northern Norway) with reference to the corresponding figures in the main text representing provenance P121 (early timing of budburst, originating from Germany). Figure B6 includes information on a) P121 and b) P122-123.

| **Provenance P122** | **Provenance P123** |
| --- | --- |
| 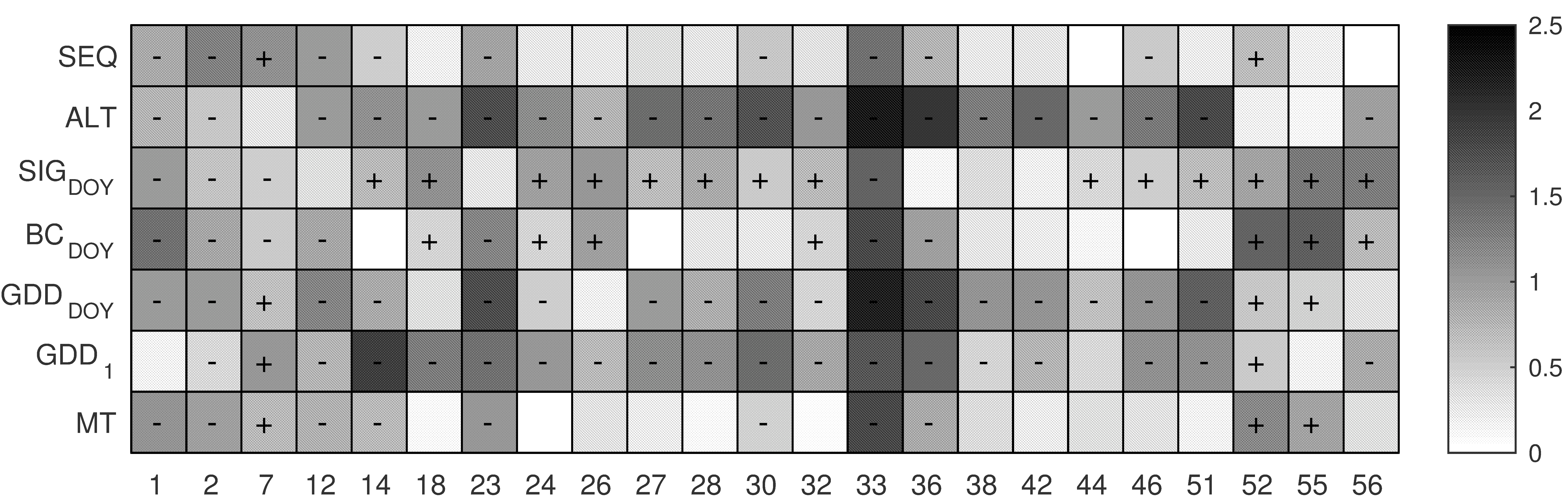 | 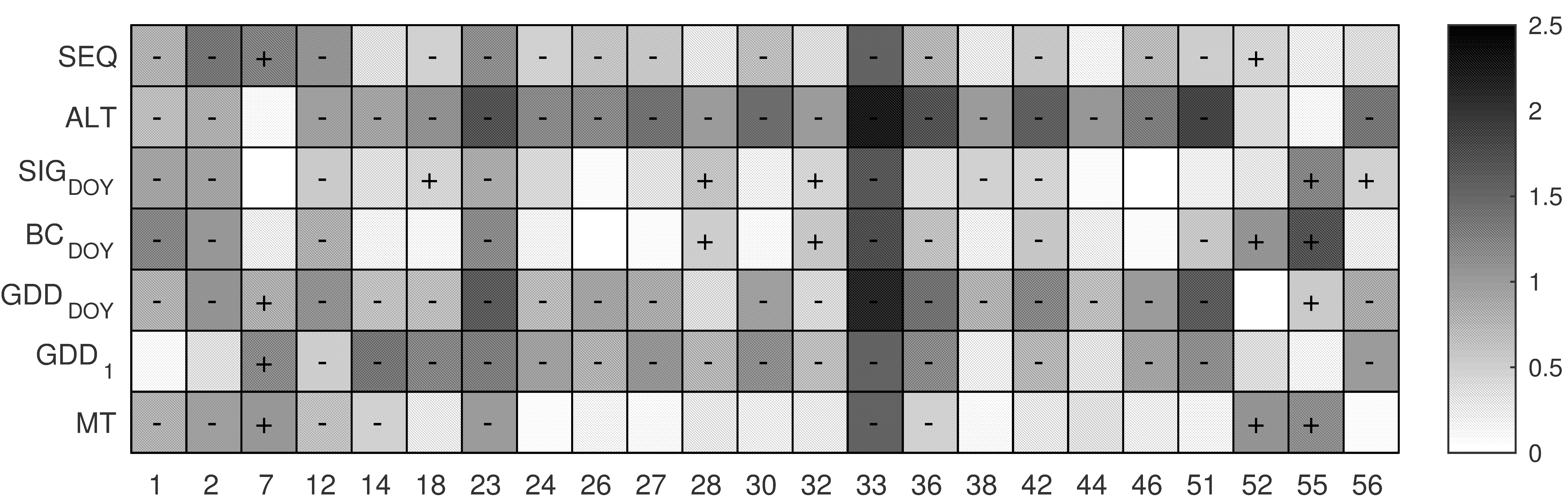 |

**Figure B1 (corresponding to Fig. 2)** Site-specific model accuracy for provenance P122 and P123, comparing distributions of budburst observations and simulations for 1971-2000, aggregated for each combination of model class (along y-axis) and International Phenological Garden (along x-axis). The grey-scale display absolute values of standardised average differences between sum of ranks of observations and simulations (Kruskal-Wallis test, α = 0.05, df = 1). Significant differences indicate that the predictive distribution (on average) includes earlier (“-”) or later (“+”) budburst than the observed distribution.

| **Provenance P122** | **Provenance P123** |
| --- | --- |
| 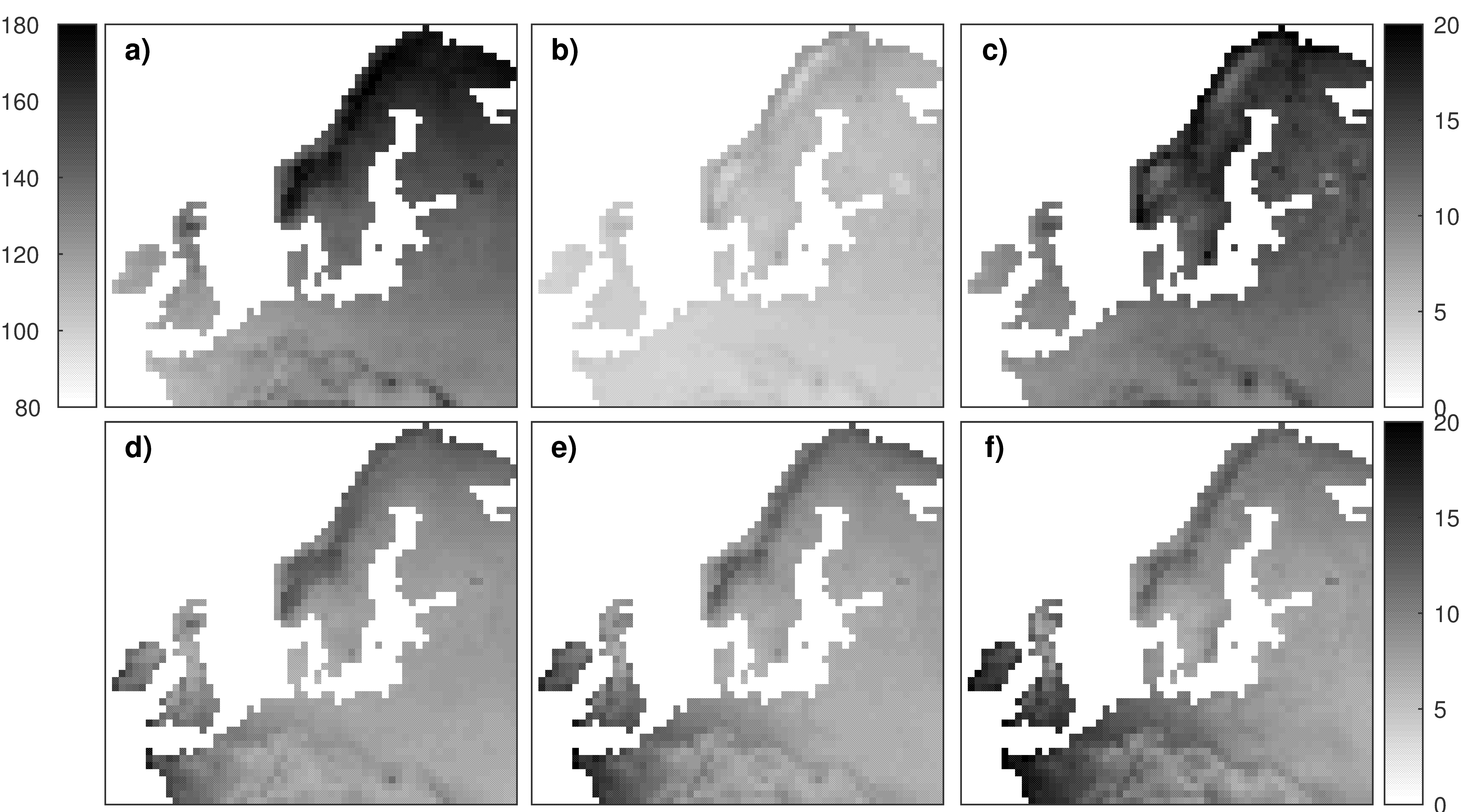 | 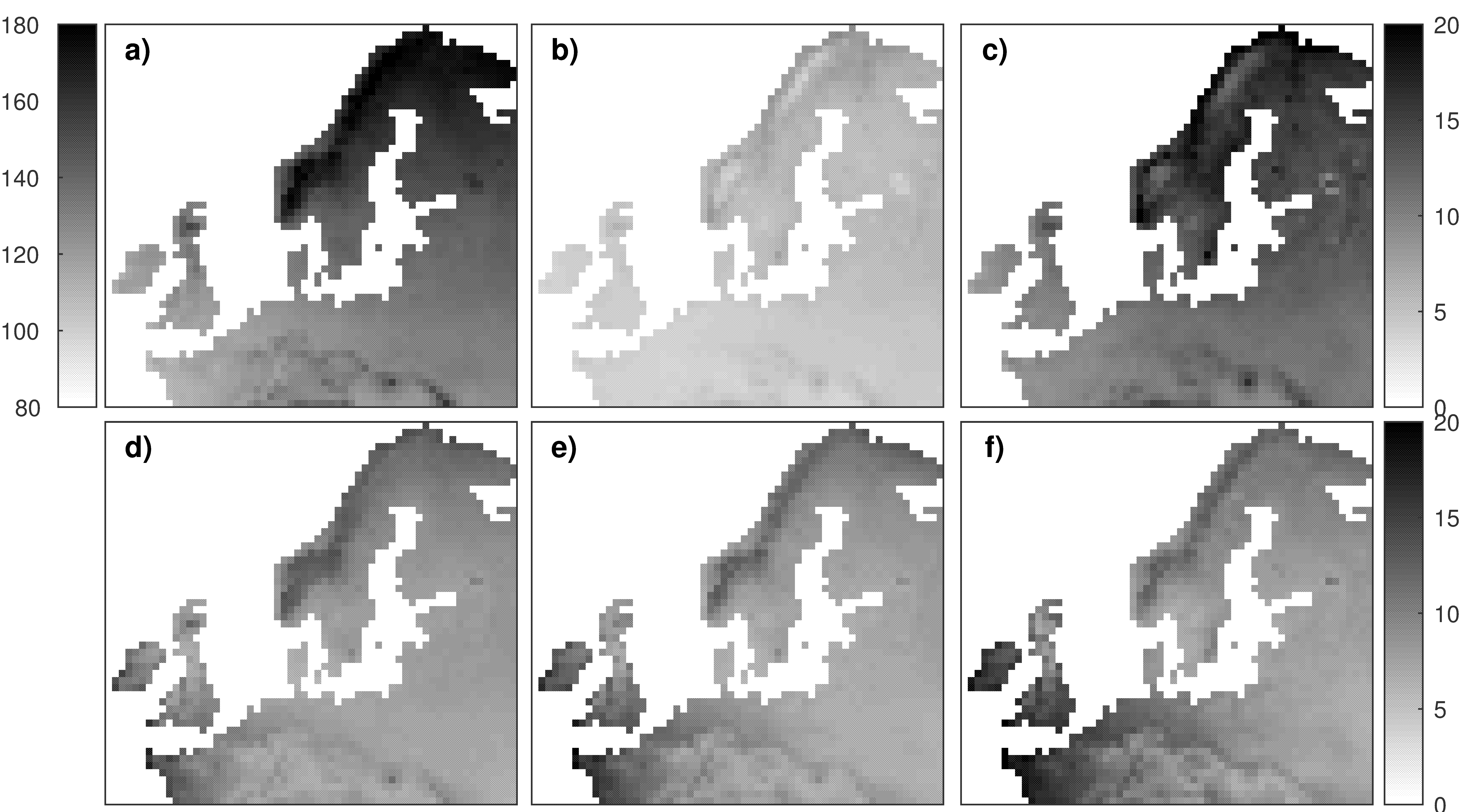 |

**Figure B2 (corresponding to Fig. 4):** Spatial variation in budburst simulations for provenance P122 and P123. a) Grid-cell ensemble means (day of year) in 1971-2000, b) average change (number of days) from 1971-2000 to 2011-2040, c) average change (number of days) from 1971-2000 to 2051-2080. The lower panels show the standard deviations for each time period, d) 1971-2000, e) 2011-2040, and f) 2051-2080. The grid-cell ensemble means are based on the initial conditions IPGAll, model classes MT, GDD_1_, GDD_DOY_, BC_DOY_, SIG_DOY_, and ALT, and boundary conditions CanESM2, CERFACS, IPSL, NorESM1 and GFDL (see Section 3.1 for subset selection).

| **November-January** | **February-April** |
| --- | --- |
| 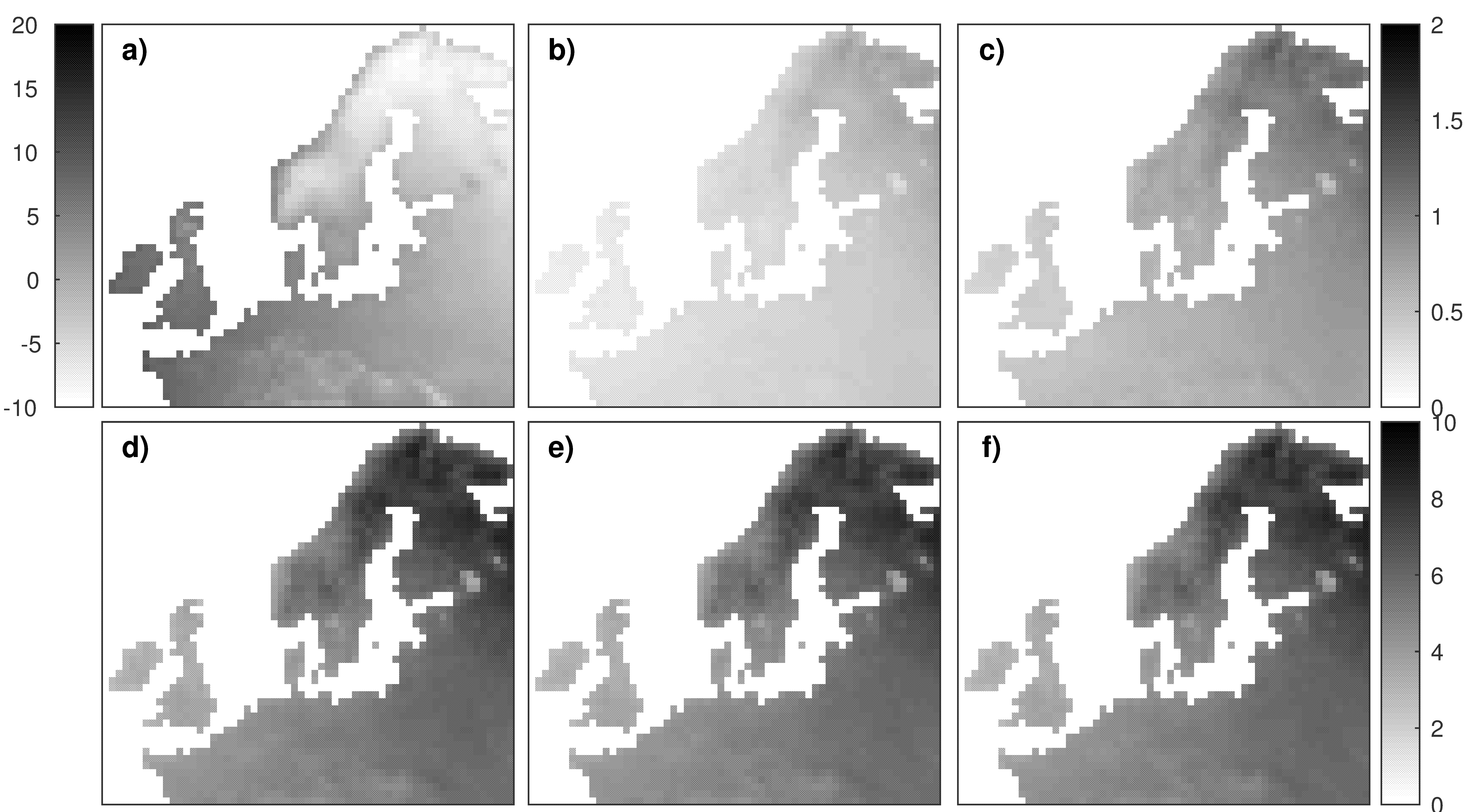 | 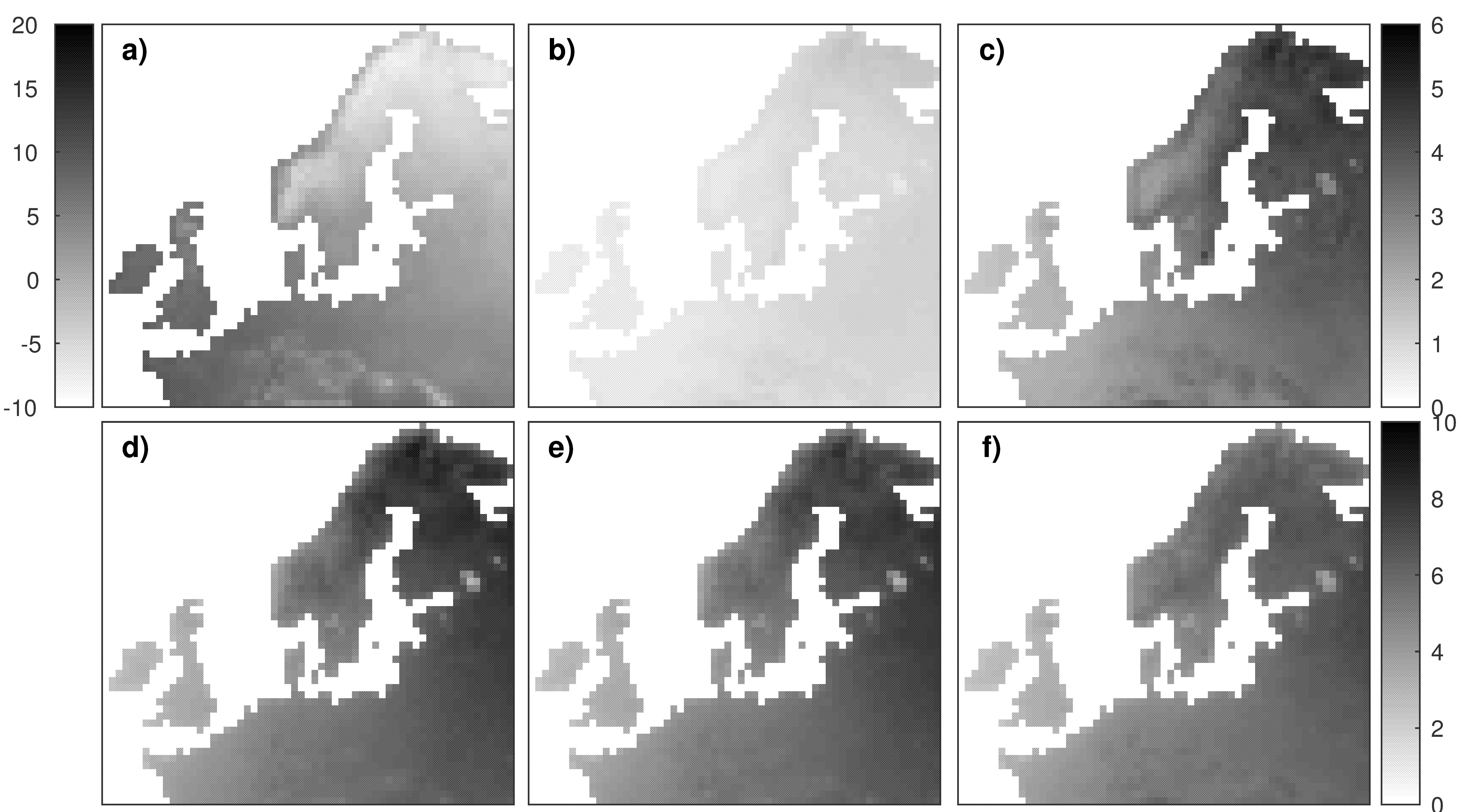 |

**Figure B3:** Ensemble mean temperature (climate model data from RCA4 used in dynamical downscaling of five global climate models; CanESM2, CERFACS, IPSL, NorESM1 and GFDL), a) mean (°C) in 1971-2000, b) average increase (°C) from 1971-2000 to 2011-2040, c) average increase (°C) from 1971-2000 to 2051-2080. The lower panels show the standard deviations (°C) for each time period, d) 1971-2000, e) 2011-2040, and f) 2051-2080.

| **Provenance P122** | **Provenance P123** |
| --- | --- |
| 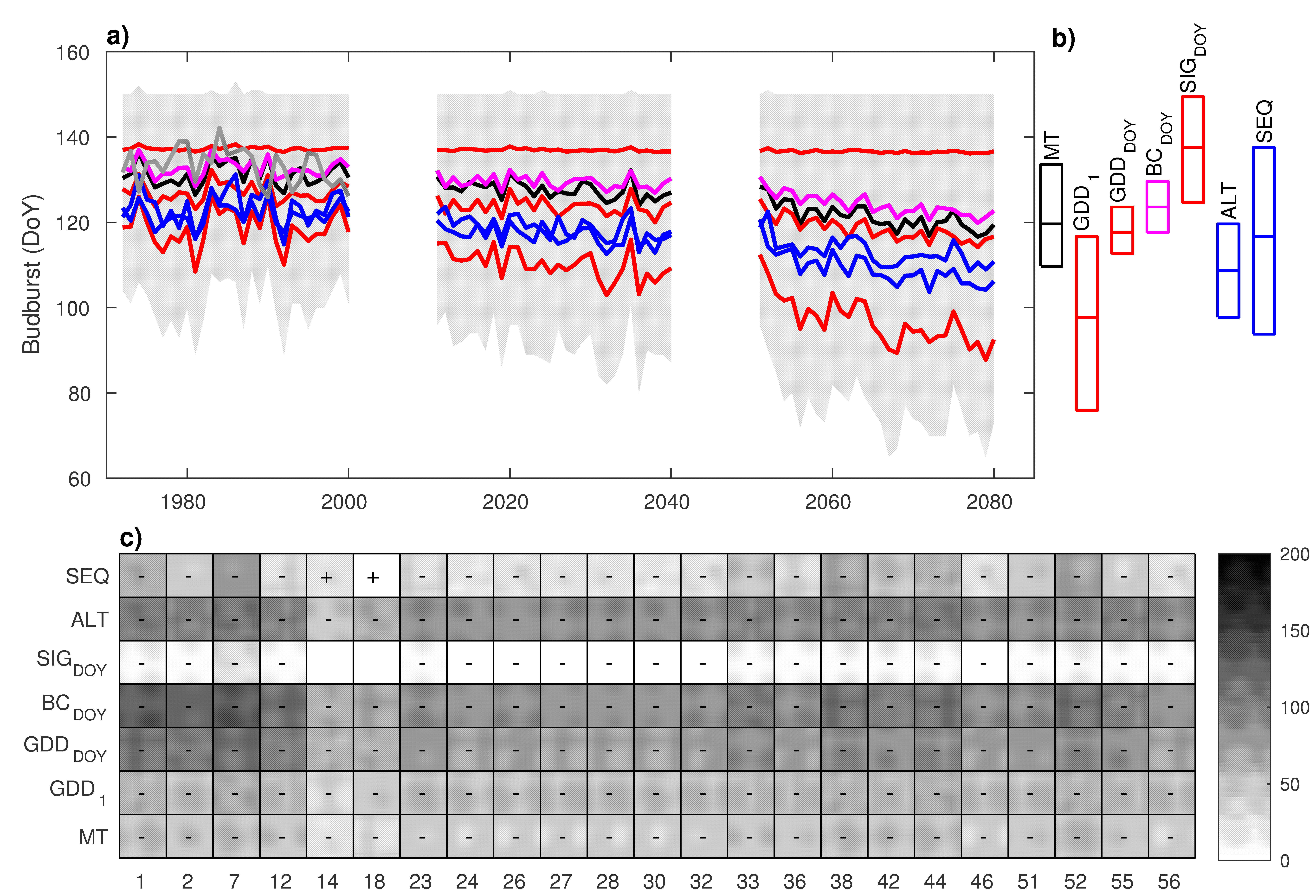 | 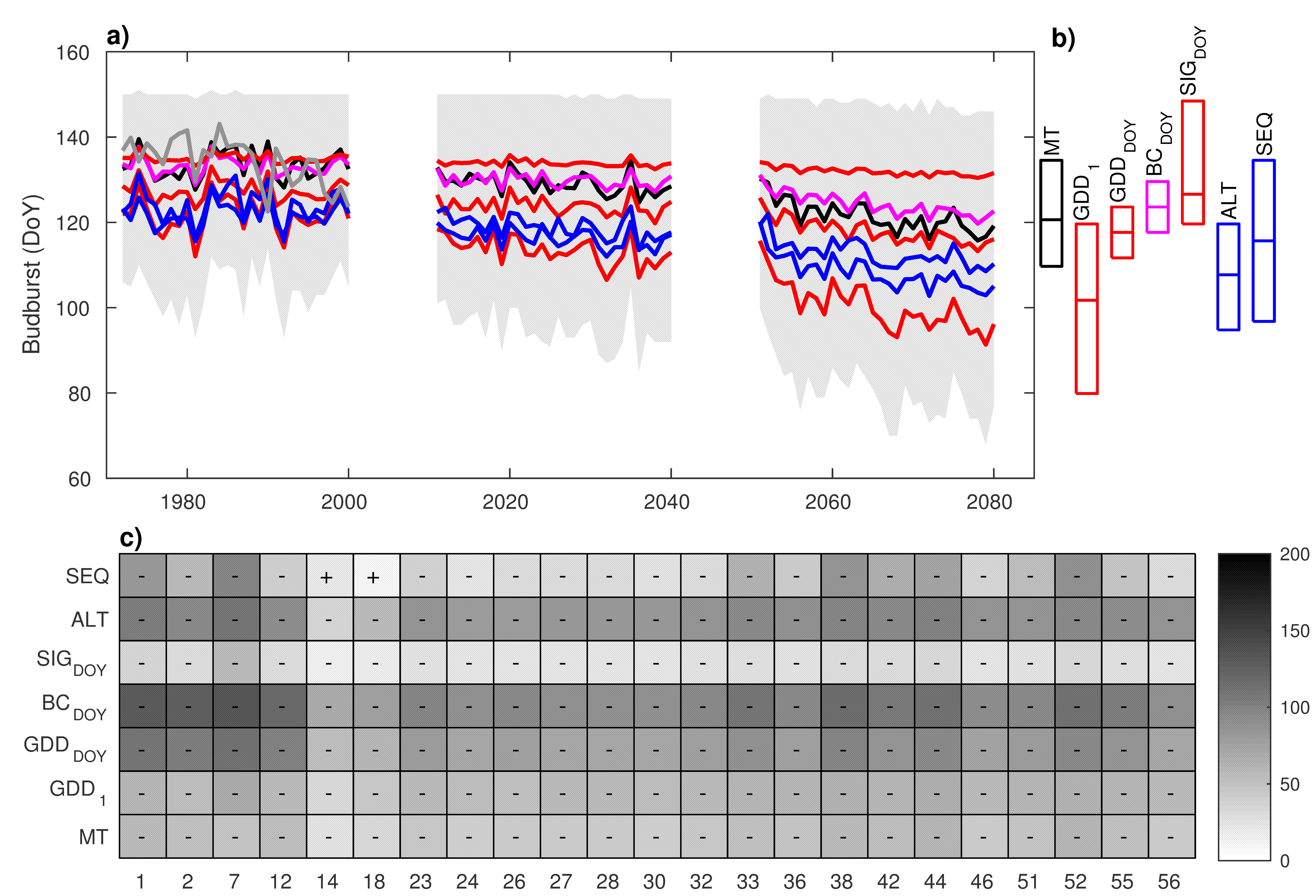 |

**Figure B4 (Fig. 5):** Three panels displaying the results of phenology model projections for provenance P122 and P123 in relation to a) interannual variation across the study region for the three selected time periods, b) variation among phenology models for the last time period, and c) variations in the strength of the climate change signal among models and International Phenological Gardens. The colour in a) and b) group the models in relation to the physiological processes included: empirical (black), forcing (red), forcing modified by photoperiod (magenta), and chilling and forcing (blue), with the line representing the mean and the box the 25th and 75th percentile. In c) the colour display absolute values of standardised average differences between sum of ranks of distributions aggregated for each combination of model class (along y-axis) and International Phenological Garden (along x-axis) (Kruskal-Wallis test, α = 0.05, df = 1). Significant differences (negative "-" or positive "+") in simulations between 1971-2000 and 2051-2080 (Kruskal-Wallis test, α = 0.05, df = 1).

| **Provenance P122** | **Provenance P123** |
| --- | --- |
| 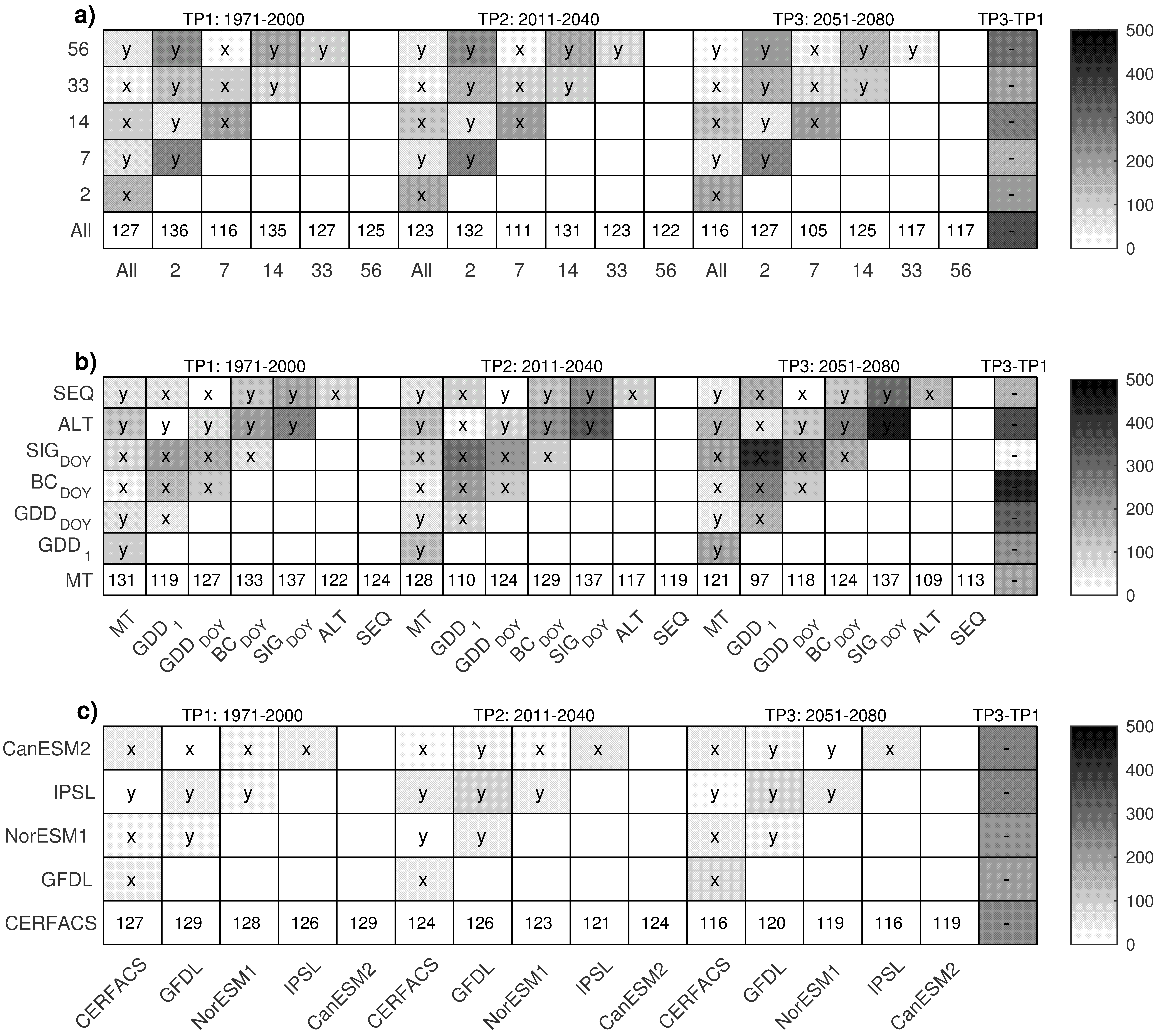 | 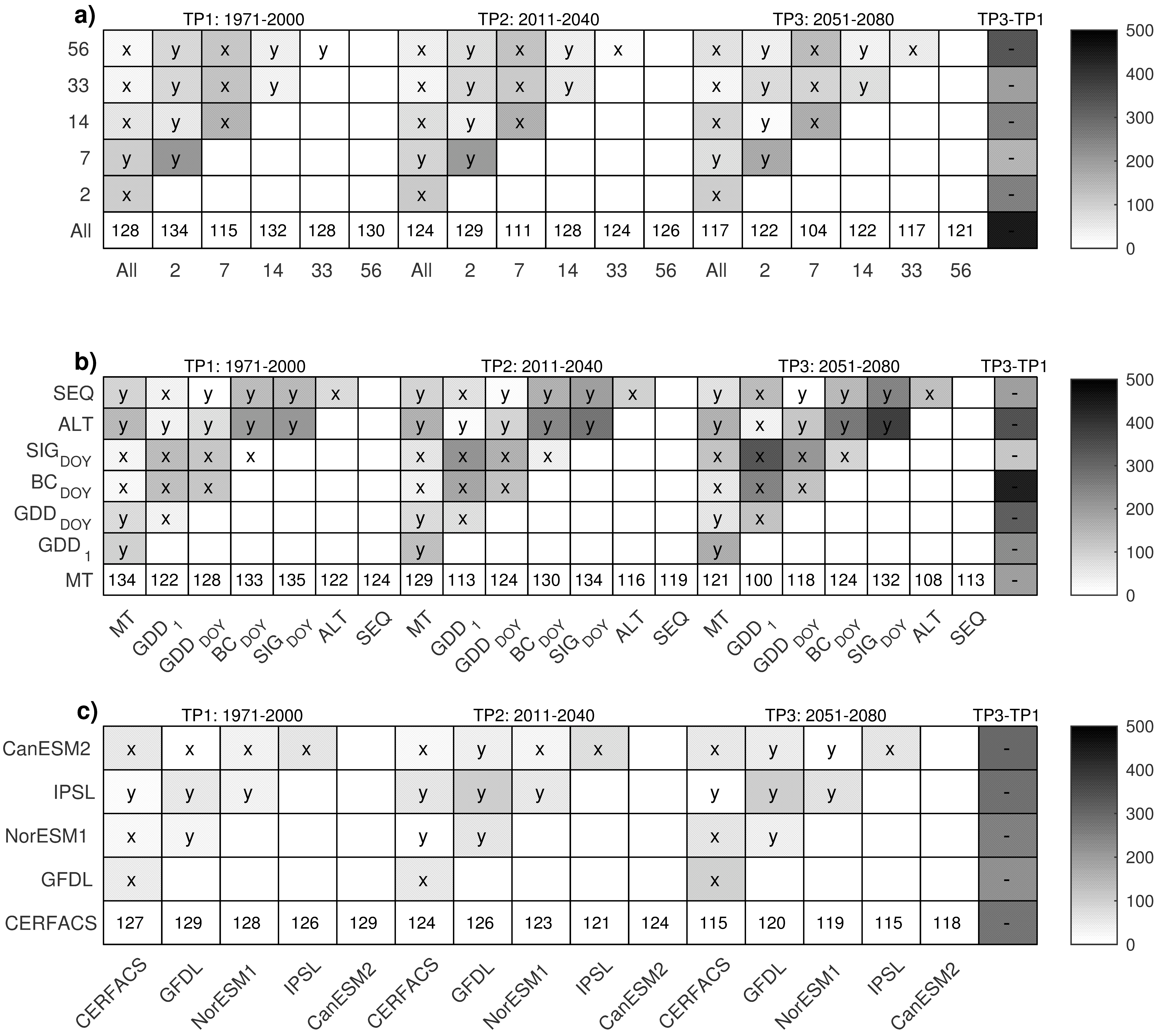 |

**Figure B5 (corresponding to Fig. 6):** The relative contribution of initial conditions (IC), model classes (MC), and boundary conditions (BC) to uncertainties in the model projections for each time period. Differences in budburst simulations for provenance P122 and P123 among cumulative density functions across a) IC, b) MC and c) BC. The distribution averages are presented along the x-axis. The grey-scale display absolute values of standardised average differences between sum of ranks of the distributions (Kruskal-Wallis test with Bonferroni-corrected p-values, α = 0.05, a) df = 6, b) df = 6, c) df = 5). Significant differences in the post hoc pairwise comparison indicate the distribution that (on average) include earlier budburst (x-axis “x” or y-axis “y”). The far right panels indicate significant differences (negative "-" or positive "+") in simulations between 1971-2000 and 2051-2080 for the distribution on the y-axis (Kruskal-Wallis test, α = 0.05, df = 1).

| **Provenance P121** |
| --- |
| 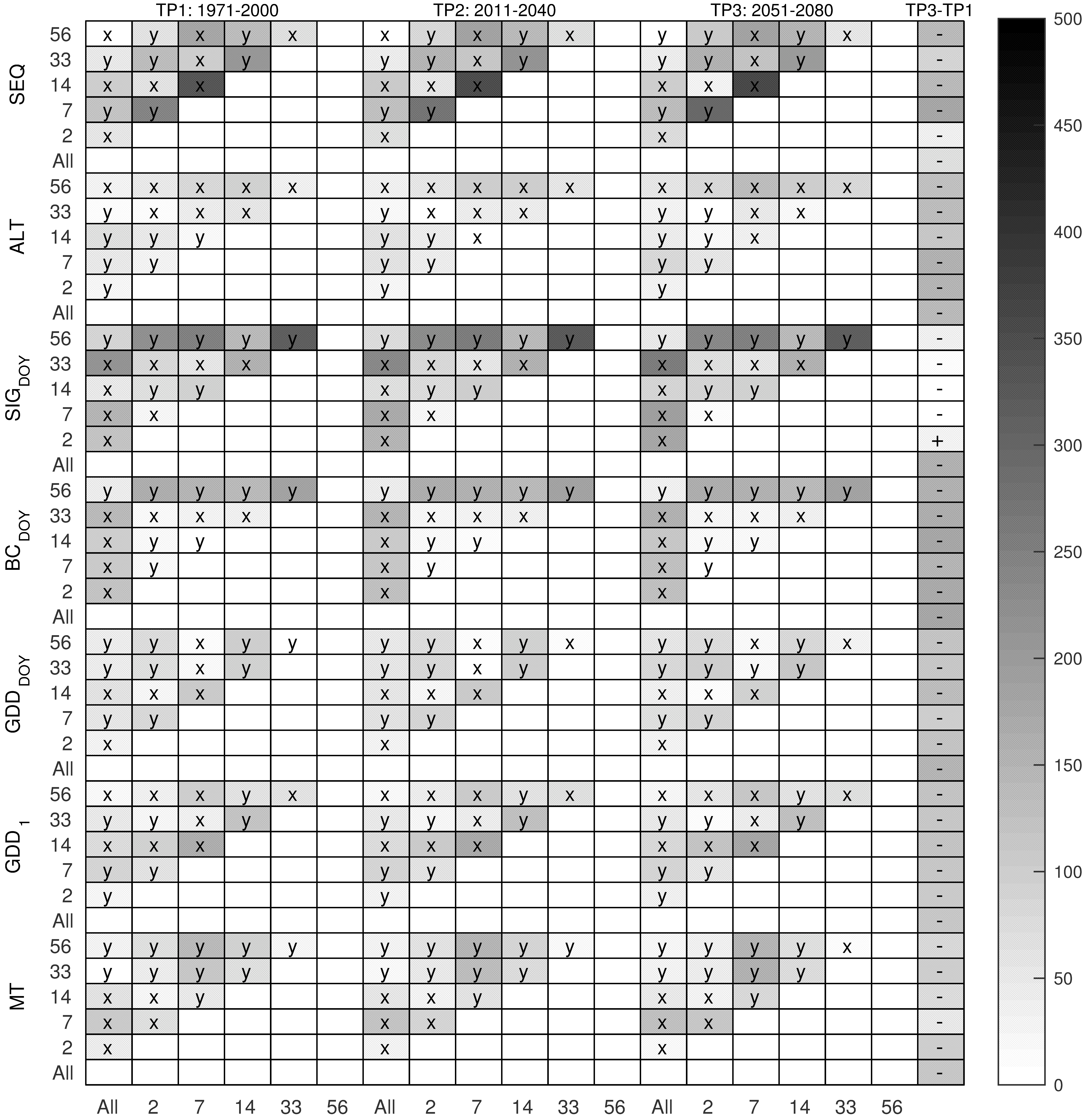 |

**Figure B6a:** Model class sensitivity to initial conditions for each time period. Differences in budburst simulations for provenance P122 and P123 among cumulative density functions of model class (along y-axis) and International Phenological Garden (along x-axis). The grey-scale display absolute values of standardised average differences between sum of ranks of the distributions (Kruskal-Wallis test with Bonferroni-corrected p-values, α = 0.05, df = 6). Significant differences in the post hoc pairwise comparison indicate the distribution that (on average) include earlier budburst (x-axis “x” or y-axis “y”). The far right panels indicate significant differences (negative "-" or positive "+") in simulations between 1971-2000 and 2051-2080 for the distribution on the y-axis (Kruskal-Wallis test, α = 0.05, df = 1).

| **Provenance P122** | **Provenance P123** |
| --- | --- |
| 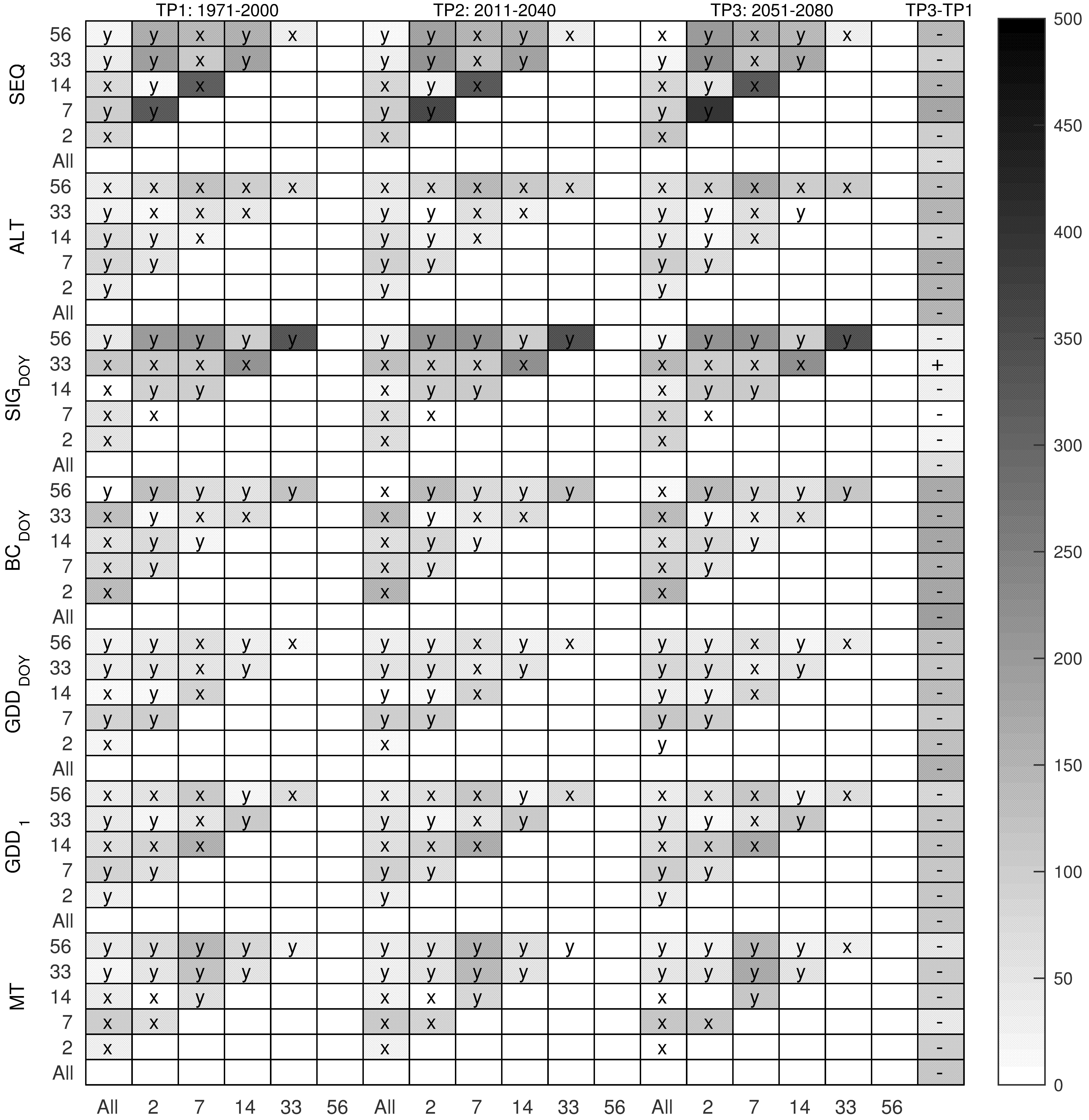 | 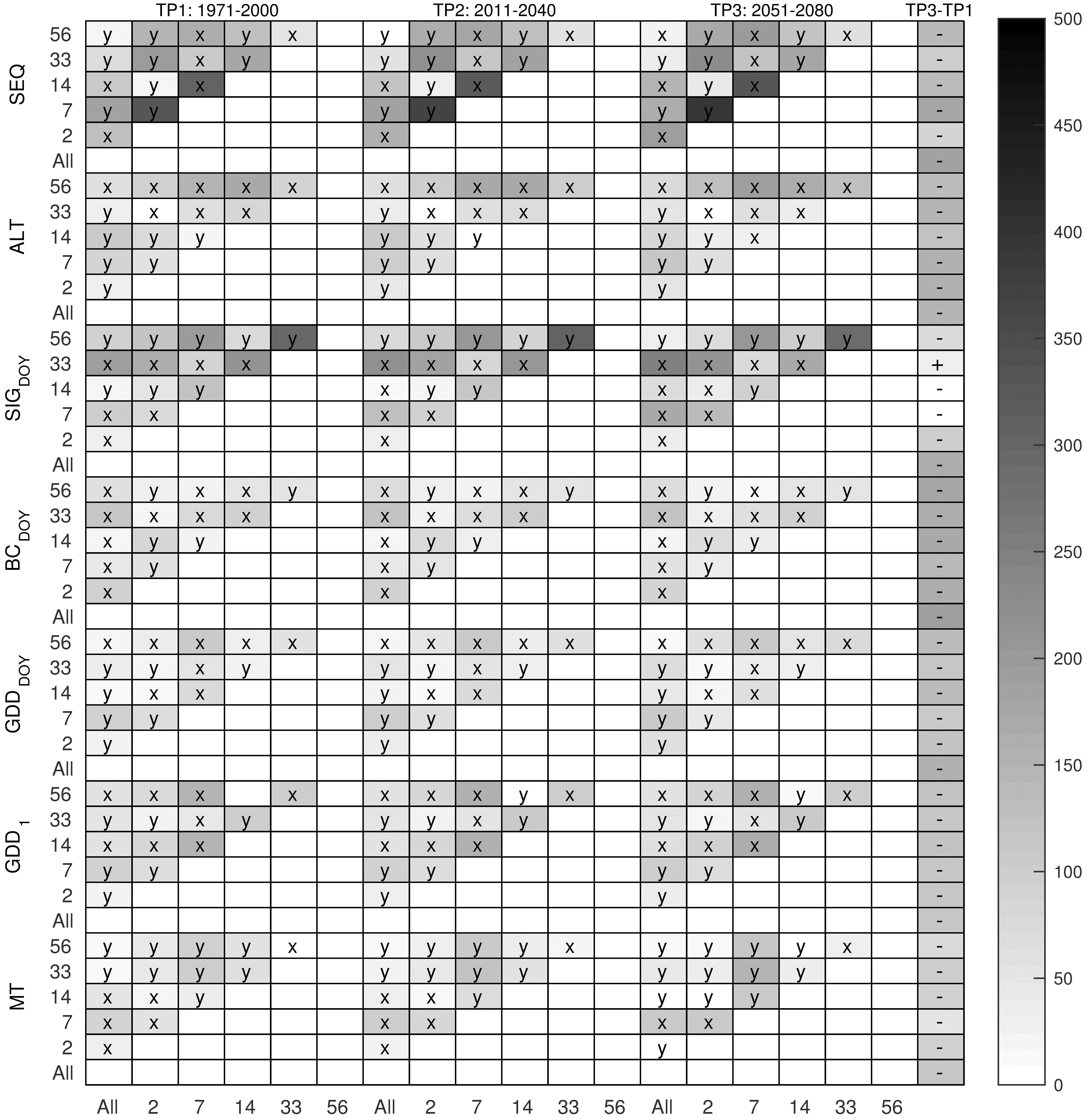 |

**Figure B6b:** Model class sensitivity to initial conditions for each time period. Differences in budburst simulations for provenance P122 and P123 among cumulative density functions of model class (along y-axis) and International Phenological Garden (along x-axis). The grey-scale display absolute values of standardised average differences between sum of ranks of the distributions (Kruskal-Wallis test with Bonferroni-corrected p-values, α = 0.05, df = 6). Significant differences in the post hoc pairwise comparison indicate the distribution that (on average) include earlier budburst (x-axis “x” or y-axis “y”). The far right panels indicate significant differences (negative "-" or positive "+") in simulations between 1971-2000 and 2051-2080 for the distribution on the y-axis (Kruskal-Wallis test, α = 0.05, df = 1).
